# Advances in Polymeric Semiconductors for Next-Generation Electronic Devices

**DOI:** 10.3390/polym17233174

**Published:** 2025-11-28

**Authors:** Ju Won Lim

**Affiliations:** Division of Semiconductor and Electronics Engineering, Hankuk University of Foreign Studies, Yongin 17035, Republic of Korea; jwlim@hufs.ac.kr

**Keywords:** polymeric materials, semiconductor materials, semiconductor devices, material properties, physical mechanism, optoelectronics, neuromorphic devices, phototransistors

## Abstract

Polymeric semiconductors have rapidly evolved from early conductive polymers, such as polyacetylene, to high-performance donor–acceptor copolymers, offering a unique combination of mechanical flexibility, solution processability, and tunable optoelectronic properties. These advancements have positioned polymeric semiconductors as versatile materials for next-generation electronics, including wearable, stretchable, and bio-integrated devices, IoT systems, and soft robotics. In this review, we systematically present the fundamental principles of polymeric semiconductors, including electronic structure, charge transport mechanisms, molecular packing, and solid-state morphology, and elucidate how these factors collectively govern device performance. We further discuss recent advances in synthesis strategies, thin-film processing techniques, molecular doping, and interface engineering, emphasizing their critical roles in improving operational stability, charge-carrier mobility, and energy efficiency. Key applications—such as organic photovoltaics, field-effect transistors, neuromorphic devices, and memristors—are analyzed, with a focus on the intricate structure–property–performance relationships that dictate functionality. Finally, we highlight emerging directions and scientific innovations, including sustainable and degradable polymers, hybrid and two-dimensional polymer systems, and novel strategies to enhance device stability and performance. By integrating fundamental polymer science with device engineering, this review provides a comprehensive, structured, and forward-looking perspective, identifying knowledge gaps and offering insights to guide future breakthroughs and the rational design of high-performance, multifunctional, and environmentally responsible polymeric electronic devices.

## 1. Introduction

The rapid evolution of modern electronics has been driven by an enduring pursuit of materials that are not only electrically efficient but also mechanically adaptive, lightweight, and environmentally sustainable [1,2,3]. In this context, polymeric semiconductors have emerged as a transformative class of materials, offering a compelling alternative to conventional inorganic and small-molecule semiconductors. Distinguished by their intrinsic flexibility, chemical tunability, and solution processability, conjugated polymers uniquely combine electronic functionality with mechanical compliance—an unprecedented synergy that opens the door to truly flexible, stretchable, and even bio-integrated electronic systems. Their ability to be printed or coated from solution onto diverse substrates using low-temperature, additive, and large-area fabrication techniques redefines how electronics can be manufactured and utilized in the future [4,5,6].

Unlike rigid crystalline semiconductors such as silicon or gallium arsenide, polymeric semiconductors possess soft mechanical properties and low modulus values that allow them to conform to dynamic, curved, or deformable surfaces without compromising electrical performance [7]. This feature is particularly advantageous for next-generation applications where electronics must intimately interface with biological tissues or flexible platforms. Especially, the molecular architecture of conjugated polymers provides another layer of versatility: by rationally designing donor and acceptor units, modulating side-chain structures, or introducing noncovalent interactions, researchers can precisely tune the energy bandgap, energy-level alignment, and charge-transport characteristics [8,9]. Such molecular-level control enables the design of materials optimized for specific device functions—whether for efficient charge transport in organic field-effect transistors (OFETs) [10,11], balanced exciton dynamics in organic photovoltaics (OPVs) [12,13], neuromorphic devices [14,15], and memristors [16,17]. Advances in polymer design have increasingly emphasized environmental and operational stability, addressing long-standing challenges related to degradation and morphological instability.

Beyond their outstanding materials versatility, polymeric semiconductors are central to the vision of next-generation electronics—systems that emphasize adaptability, sustainability, and human-centered design. The emergence of wearable and bio-integrated electronics demands materials that can maintain stable electrical function under continuous bending, stretching, or contact with physiological environments. Polymeric semiconductors, by virtue of their inherent flexibility and biocompatibility, have proven particularly suitable for such applications, enabling conformable health monitors, skin-like sensors, and implantable bioelectronic interfaces [18]. Simultaneously, the global expansion of the Internet of Things (IoT) has accelerated the need for lightweight, disposable, and energy-efficient electronic components that can be distributed ubiquitously in the environment. The compatibility of conjugated polymers with printing technologies allows the fabrication of low-cost, large-area, and even biodegradable electronics—an essential step toward sustainable and resource-efficient manufacturing paradigms.

At the device level, polymeric semiconductors have demonstrated exceptional versatility across a wide range of electronic and optoelectronic platforms. In OPVs, rational control of donor–acceptor architectures and nanoscale phase morphology has enabled efficient exciton generation, dissociation, and charge transport, leading to continuous improvements in power conversion efficiency [19]. OFETs have benefited from advances in backbone planarity, side-chain engineering, and solid-state ordering, achieving high carrier mobilities while preserving mechanical compliance and optical transparency [20]. Meanwhile, the intrinsic coupling of ionic and electronic transport in conjugated polymers has spurred the emergence of neuromorphic and memristive devices capable of emulating synaptic plasticity, thereby bridging electronics and biology in unprecedented ways [21]. Together, these diverse demonstrations highlight how the molecular design principles governing polymeric semiconductors can be leveraged to realize multifunctional, adaptive, and energy-efficient devices that underpin the vision of soft and intelligent electronics.

This review aims to provide a comprehensive and critical overview of recent progress in polymeric semiconductors, highlighting the fundamental design principles, charge-transport mechanisms, processing strategies, and device applications that have propelled the field toward next-generation electronics. We emphasize how molecular design, structural control, and processing innovations collectively determine the electrical, mechanical, and environmental performance of polymeric semiconductors. By integrating insights across materials chemistry, condensed matter physics, and electronic engineering, this review seeks to elucidate the key structure–property–function relationships that govern polymeric semiconductors and to identify the scientific frontiers that will guide their evolution. Ultimately, this work aims to position polymeric semiconductors not merely as functional materials, but as a cornerstone of the technological transformation toward soft, sustainable, and human-adaptive electronics.

## 2. Fundamentals of Polymeric Semiconductors

Polymeric semiconductors are a unique class of organic materials whose electrical properties arise from the presence of extended π-conjugation along their molecular backbones. Unlike conventional inorganic semiconductors, where charge carriers move through a rigid crystal lattice, polymeric semiconductors consist of flexible chains with alternating single and double bonds, enabling the delocalization of π-electrons across the conjugated segments. This delocalization gives rise to valence and conduction bands separated by an optical bandgap, which can be tailored through molecular design. The intrinsic electronic structure—defined by the energy levels of the highest occupied and lowest unoccupied molecular orbitals (HOMO and LUMO)—determines the material’s ability to absorb light, generate excitons, and transport charge carriers [8]. Chemical modification of the polymer backbone, such as incorporating electron-rich or electron-deficient units, allows precise tuning of these energy levels to optimize alignment with electrodes or other layers in optoelectronic devices.

Charge transport in polymeric semiconductors is inherently influenced by their molecular ordering and the interplay between intrachain and interchain pathways [22]. Along a single polymer chain, charge carriers can move efficiently through delocalized orbitals, but in the solid state, transport is often limited by interchain hopping across weak van der Waals or π–π interactions [22,23]. The semicrystalline nature of most conjugated polymers results in heterogeneous charge transport, where localized and delocalized states coexist. Consequently, charge mobility depends not only on intrinsic electronic structure but also on extrinsic factors such as chain packing, crystallinity, and energetic disorder. Optimizing these parameters through rational molecular design, side-chain engineering, and processing control enables efficient charge delocalization and percolation across the active layer—establishing the foundation for high-performance polymer-based transistors, solar cells, and light-emitting devices.

### 2.1. Electronic Structure and Charge Transport Mechanisms

The performance of polymeric semiconductors in electronic and optoelectronic devices is fundamentally governed by their electronic structure and charge transport characteristics. Understanding how molecular architecture, conjugation length, and interchain organization affect charge generation, delocalization, and mobility is critical for optimizing device functionality. In conjugated polymers, delocalized π-electrons form the basis of semiconducting behavior, allowing for efficient charge transport through extended π–π interactions and controlled bandgap engineering.

#### 2.1.1. π–Conjugation, Bandgap Formation, and Delocalization

Conjugated polymers consist of alternating single and double bonds along their backbone, resulting in overlapping p-orbitals and the formation of a delocalized π-electron system. This delocalization gives rise to the valence and conduction bands, analogous to inorganic semiconductors, separated by an optical bandgap (*E*_g_). The bandgap magnitude is primarily determined by the extent of π-conjugation and the electron-withdrawing or -donating nature of the constituent monomer units. Increased conjugation length and planar backbone geometry enhance orbital overlap and reduce the bandgap, thereby improving charge delocalization and absorption in the visible to near-infrared regions. Conversely, structural torsion and backbone disorder disrupt conjugation, localizing charge carriers and decreasing mobility.

**Figure 1:**
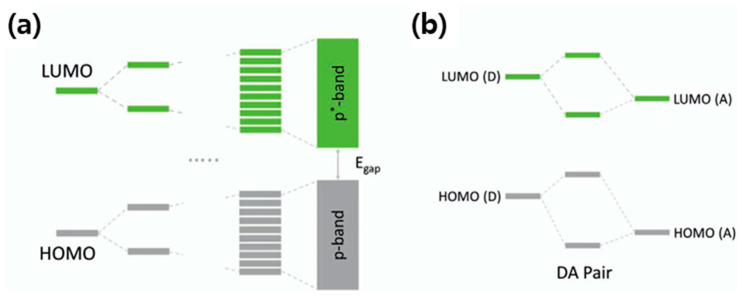
(**a**) The band gap of organic semiconductors as affected by their conjugation length. The dotted lines indicate the progression from discrete molecular orbitals to extended band-like states as conjugation length increases, while the asterisk (*) indicates an antibonding orbital. The green levels and bands represent the LUMO and π*-band states, while the gray levels and bands represent the HOMO and π-band states. (**b**) HOMO–LUMO splitting in covalently bound donor–acceptor (DA) pairs. Green denotes LUMO levels and gray denotes HOMO levels of the donor (D) and acceptor (A) units, respectively. The dotted lines indicate electronic coupling between the donor and acceptor orbitals. This figure is adapted from ref. [24] (reproduced under terms of the CC-BY license).

Figure 1 illustrates the electronic structures and energy level interactions in conjugated polymer systems. In Figure 1a, the formation of delocalized electronic bands in conjugated polymers is shown, where the discrete molecular orbitals—HOMO (highest occupied molecular orbital) and LUMO (lowest unoccupied molecular orbital)—broaden into continuous π and π* bands, respectively, as individual molecular units couple through conjugation. The energy difference between these bands defines the *E*_g_, which governs the optical and electronic properties of the polymer semiconductor. Panel (b) depicts the energy level alignment in a donor–acceptor (D–A) pair system, where the HOMO and LUMO levels of the donor and acceptor interact to create new hybridized frontier orbitals. This donor–acceptor interaction leads to intramolecular charge transfer and bandgap tuning, which are key strategies for optimizing charge separation and electronic performance in polymeric semiconductors.

#### 2.1.2. HOMO–LUMO Levels and Bandgap Tuning

The Highest Occupied Molecular Orbital (HOMO) and Lowest Unoccupied Molecular Orbital (LUMO) define the frontier energy levels of polymeric semiconductors and determine their ability to donate or accept charge carriers. Chemical modification of the polymer backbone enables precise control over these energy levels and, consequently, the bandgap. D–A copolymers, in particular, allow for systematic tuning by alternating electron-rich and electron-deficient units along the chain [25]. This approach narrows the bandgap through intramolecular charge transfer interactions and aligns energy levels with adjacent materials in devices such as OPVs and OFETs. Moreover, substituents on the backbone or side chains can influence the ionization potential and electron affinity, thereby optimizing charge injection and transport at interfaces.

#### 2.1.3. Intrachain vs. Interchain Charge Transport

**Figure 2:**
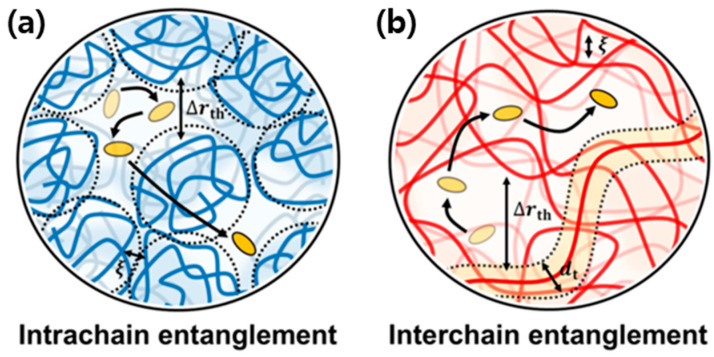
Schematic of (**a**) intrachain and (**b**) interchain entangled polymer networks. Blue and red strands represent individual polymer chains in the intrachain and interchain entanglement regimes, respectively. The dotted lines outline the corresponding confining regions within each network. Arrows indicate the direction of probe motion, and Δ*r* denotes the two-dimensional displacement between consecutive probe positions extracted from single-molecule trajectories. Here, *d*_t_, and *ξ* denote the tube diameter and correlation length, respectively. Reprinted figure with permission from ref. [26]. Copyright (2025) American Chemical Society.

As shown in Figure 2, charge transport in conjugated polymers proceeds through two primary pathways: intrachain transport, which occurs along the covalently bonded polymer backbone, and interchain transport, which takes place between adjacent chains. Intrachain charge migration is generally more efficient, as it benefits from the extended π-conjugation and strong covalent bonding that enable delocalization of charge carriers over multiple repeating units. This quasi-one-dimensional transport mechanism is characterized by high carrier mobility along the backbone and is often limited by conformational defects or torsional disorder that interrupt conjugation.

In contrast, interchain transport depends on noncovalent π–π stacking interactions and van der Waals forces between polymer chains. Because these interactions are relatively weak, interchain charge transfer is inherently slower and highly sensitive to molecular packing, crystallinity, and structural disorder. The efficiency of long-range charge percolation through the bulk film therefore depends on achieving an optimal balance between intrachain delocalization and interchain electronic coupling. Disruptions in chain alignment or variations in interchain distance can introduce energetic barriers that localize carriers and impede transport.

To enhance charge carrier mobility, a variety of morphological control strategies have been developed. Side-chain engineering is a particularly powerful approach, allowing fine-tuning of polymer solubility, backbone planarity, and intermolecular packing [27]. Additionally, thermal annealing and solvent vapor treatment are commonly employed post-processing techniques that promote the formation of ordered lamellar structures and improve π–π stacking coherence across the film. Together, these molecular and processing strategies minimize energetic disorder, enhance carrier delocalization, and facilitate efficient charge percolation in conjugated polymer systems used for organic electronic applications such as OFETs, OPVs, and memory devices.

#### 2.1.4. Factors Affecting Charge Carrier Mobility

Charge carrier mobility (*μ*) serves as a fundamental parameter describing how efficiently charge carriers—electrons or holes—move through a semiconducting material under an applied electric field. It directly reflects the balance between carrier delocalization and scattering processes and is governed by a complex interplay of intrinsic molecular characteristics and extrinsic morphological and environmental factors [28].

Intrinsic factors primarily arise from the polymer’s molecular structure and electronic configuration. The degree of molecular order, crystallinity, and effective conjugation length dictates the extent of π-orbital overlap and charge delocalization along and between polymer chains. Highly planar and rigid backbones promote extended conjugation and enhance intrachain transport by reducing torsional disorder and enabling efficient π–π stacking. Conversely, structural defects, chain twisting, chemical impurities, and side-chain irregularities act as localization centers or trap sites, interrupting charge transport pathways. Moreover, the energetic disorder associated with variations in molecular orientation and conformation can substantially reduce carrier coherence and limit long-range transport.

Extrinsic factors are predominantly associated with the solid-state microstructure and interfacial environment. Film morphology, degree of phase separation, and polymer chain alignment strongly influence interchain coupling and the formation of percolation pathways for charge transport. The dielectric environment also plays a crucial role: high-κ dielectric materials or optimized dielectric–semiconductor interfaces can effectively screen Coulombic interactions between charges and trap states, thereby enhancing the apparent mobility. In addition, controlling trap density—through careful film processing, molecular doping, or post-deposition annealing—can suppress deep trap states that impede transport.

Polymers exhibiting high regioregularity and narrow molecular weight distribution typically achieve improved packing and reduced energetic disorder, resulting in remarkably high mobilities exceeding 10 cm^2^ V^−1^ s^−1^ in optimized systems [29]. Recent advances in molecular design, such as the incorporation of donor–acceptor copolymers, side-chain engineering, and noncovalent conformational locks, further demonstrate how subtle structural modifications can tune charge transport by balancing intrachain delocalization and interchain connectivity. Collectively, these insights underscore that achieving high carrier mobility requires simultaneous optimization of molecular architecture, supramolecular ordering, and device-level interfaces. The interplay between molecular structure and optoelectronic behavior is central to the design of high-performance polymeric semiconductors. Structural features such as backbone planarity, conjugation length, and side-chain engineering collectively determine the optical absorption, emission spectra, and charge transport characteristics. For example, incorporating fused aromatic rings increases backbone rigidity and enhances π-overlap, leading to red-shifted absorption and improved charge mobility. Conversely, bulky or branched side chains, while beneficial for solubility and film formation, can disrupt π–π stacking and reduce interchain transport. Balancing these competing structural factors is thus essential for achieving optimal performance across diverse applications, from transistors and sensors to light-emitting and photovoltaic devices. Ultimately, molecular design strategies that synergistically tune the electronic structure and solid-state morphology offer the most promising route toward next-generation polymer electronics. From the mobility, we can derive electrical conductivity using *σ* = *nqμ*, indicating that higher mobility directly enhances charge transport efficiency in polymeric semiconductors (See Table 1) [30].

### 2.2. Charge Transport Mechanisms

Charge transport in organic semiconductors, particularly in conjugated polymers and small molecules, is a complex process governed by the interplay between molecular structure, electronic coupling, and morphological order. Unlike crystalline inorganic semiconductors where band transport dominates, organic systems exhibit diverse transport regimes depending on the degree of order and electronic delocalization. The nature of charge motion—whether localized or delocalized—is determined by parameters such as molecular packing, energetic disorder, and temperature. Fundamentally, charge carriers in conjugated systems migrate through a combination of intrachain transport, occurring along the covalently bonded polymer backbone, and interchain transport, which relies on π–π stacking and van der Waals interactions between adjacent molecules. The efficiency of these two pathways is dictated by the molecular alignment and degree of energetic disorder. Consequently, the overall charge transport behavior emerges from the competition and cooperation between localized hopping events and extended band-like motion, leading to a temperature- and morphology-dependent mobility landscape.

#### 2.2.1. Hopping vs. Band-like Transport

**Figure 3:**
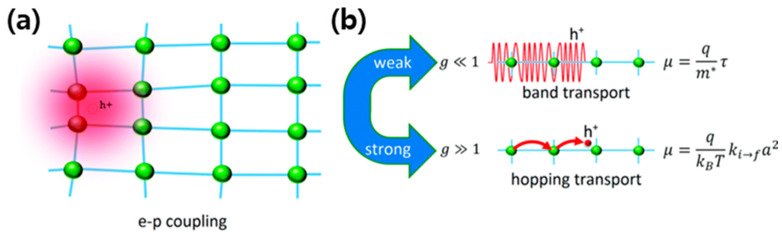
(**a**) Schematic illustration of polaron formation induced by electron–phonon (e–p) coupling, highlighting how increasing coupling strength localizes the charge carrier (h^+^) and transitions the system from a delocalized band-like regime to a localized hopping regime. (**b**) Comparison of the weak (*g* ≪ 1) and strong (*g* ≫ 1) coupling limits. The curved arrow indicates the transition between regimes, and the red shading highlights carrier localization. The asterisk (*) denotes the effective mass. Reprinted figure with permission from ref. [31]. Copyright (2017) The Royal Society of Chemistry.

Two primary regimes of charge transport are commonly distinguished in organic semiconductors: hopping transport and band-like transport (See Figure 3). Hopping transport dominates in systems with substantial energetic disorder and weak electronic coupling between sites. In this regime, charge carriers are localized on molecular sites and move via thermally activated hops assisted by phonons. The carrier mobility (*μ*) follows an Arrhenius-type temperature dependence, typically decreasing exponentially with decreasing temperature. Models such as the Miller–Abrahams and Marcus theory frameworks have been widely applied to describe this process, where the rate of charge transfer depends on both the energetic barrier and the degree of wavefunction overlap between localized states.

In contrast, band-like transport arises in highly ordered systems where π–π orbital overlap facilitates the delocalization of charge carriers over multiple molecular units. In this regime, the mobility increases as temperature decreases—opposite to the hopping case—due to reduced phonon scattering. Experimental evidence of band-like transport has been observed in single crystals and highly crystalline polymer films such as poly(3-hexylthiophene) (P3HT) and 2D π-conjugated frameworks. However, the coexistence of both mechanisms is often observed in practical materials, where partial delocalization occurs within ordered domains while hopping governs inter-domain transitions. The balance between these two regimes defines the upper limit of achievable mobility in organic semiconductors.

#### 2.2.2. Role of Traps and Disorder

Trapping states, often caused by impurities, chemical defects, or chain ends, further impedes carrier motion by immobilizing charges at localized energy levels within the bandgap. Trapped carriers can significantly alter device behavior, leading to hysteresis, threshold voltage shifts, and reduced operational stability. In polymer semiconductors, deep traps associated with chain kinks or oxygen-related defects are particularly detrimental. Strategies such as molecular doping, passivation of trap states, and controlled crystallization have been shown to mitigate these effects. A thorough understanding of disorder and trapping is therefore indispensable for accurately modeling transport phenomena and improving the reliability of organic electronic devices [32,33]. Energetic and structural disorder play decisive roles in determining charge transport efficiency. In organic semiconductors, disorder originates from variations in molecular packing, conformational torsions, and fluctuations in the local dielectric environment. This leads to a broad distribution of energy states, often modeled as Gaussian or exponential density of states (DOS). Charge carriers in such disordered systems tend to be localized in low-energy sites, resulting in dispersive transport and low mobility. As disorder increases, the transport pathway becomes more tortuous, reducing the effective percolation network available for conduction [34].

#### 2.2.3. Effect of Molecular Ordering and Aggregation

Molecular ordering and aggregation profoundly influence the charge transport properties of conjugated systems by modulating both intrachain and interchain electronic couplings. In well-ordered systems, planar molecular backbones promote efficient π–π stacking and enhance interchain orbital overlaps, enabling long-range charge delocalization. Crystalline domains serve as high-mobility pathways, while amorphous regions act as barriers or trap-rich interfaces that limit transport. The degree of crystallinity, domain orientation, and lamellar spacing collectively determine the anisotropy of charge mobility observed in thin films [35].

Furthermore, molecular aggregation can lead to distinct photophysical and transport behaviors, depending on the nature of the aggregation. H-aggregates, characterized by face-to-face stacking, often reduce charge mobility due to destructive interference between transition dipoles, while J-aggregates—head-to-tail aligned structures—can enhance delocalization and carrier transport. Processing conditions such as solvent selection, annealing temperature, and substrate interactions are thus critical in tailoring aggregation behavior. Recent studies using techniques like grazing-incidence X-ray scattering (GIXS) and time-resolved spectroscopy have provided direct correlations between molecular packing motifs and charge mobility. Overall, promoting favorable molecular ordering and minimizing energetic disorder remain the most effective strategies for achieving high-performance organic semiconductors.

### 2.3. Molecular Packing and Microstructure

**Figure 4:**
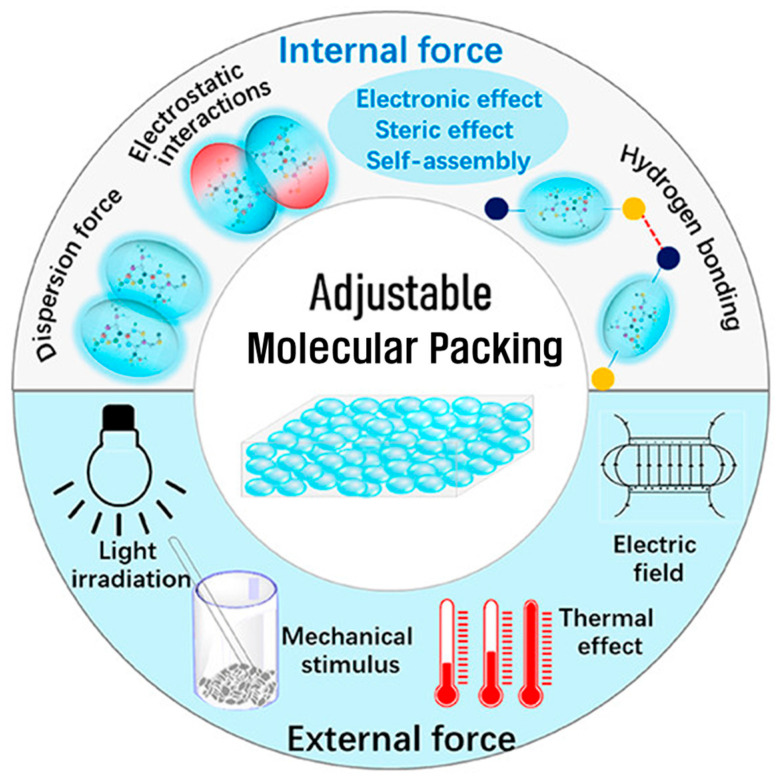
Schematic illustration of adjustable molecular packing in organic semiconductors. Molecular packing and microstructure critically govern charge transport by controlling orbital overlap and energetic disorder. Internal forces (e.g., dispersion, electrostatic, hydrogen bonding, and steric/electronic effects) and external stimuli (light, heat, mechanical, and electric fields) collaboratively tune molecular organization, thereby modulating electronic performance. Reprinted figure with permission from ref. [36] Copyright (2020) American Chemical Society.

The molecular packing and microstructure of organic semiconductors play a pivotal role in determining their electronic properties and device performance. The spatial arrangement of polymer chains or small molecules governs the degree of π–orbital overlap, the continuity of charge transport pathways, and the distribution of energetic traps [36]. As illustrated in Figure 4, molecular organization can be tuned through a combination of internal forces (e.g., dispersion, electrostatic, hydrogen-bonding, and steric or electronic effects) and external stimuli (such as light, heat, mechanical, or electric fields). This tunability underscores the central importance of packing control in optimizing charge mobility, photophysical behavior, and overall device efficiency. Moreover, structural ordering spans multiple length scales—from intrachain conformations to interchain stacking and mesoscale domain organization—reflecting the inherently hierarchical nature of organic semiconductor microstructures.

Beyond the intrinsic molecular structure, processing conditions such as solvent selection, thermal annealing, and deposition techniques strongly influence the resulting morphology. Highly ordered domains, often manifested as crystalline regions, provide quasi-continuous pathways for efficient charge transport, whereas disordered or amorphous domains introduce energetic barriers and trap states [37]. The interplay between ordered and disordered regions ultimately shapes the balance between hopping and band-like transport, emphasizing the need to characterize and engineer both micro- and nanoscale features for high-performance materials.

#### 2.3.1. Molecular Alignment and Side-Chain Engineering in π–π Stacking Control

The efficiency of charge transport in conjugated polymers is intimately linked to the degree of molecular order and the hierarchical organization of their microstructures. Parameters such as chain alignment, crystallinity, regioregularity, and side-chain architecture collectively dictate how effectively charge carriers can migrate through the organic matrix. Because charge transport in these materials occurs through a combination of intrachain delocalization and interchain hopping, the geometric arrangement of polymer backbones and the extent of π–π orbital overlap become central determinants of carrier mobility [38].

**Figure 5:**
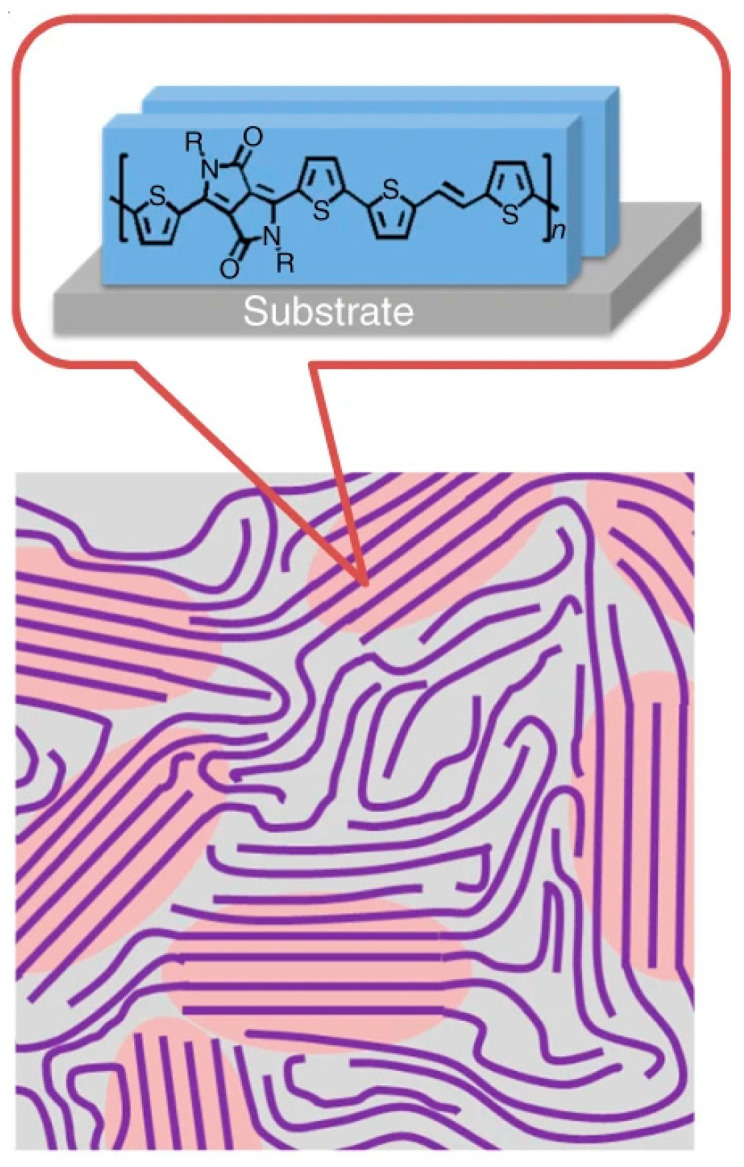
Schematic representation of a polymer thin film containing coexisting crystalline (purple) and amorphous (pink-shaded) regions. The crystalline domains consist of well-ordered lamellar structures with polymer chains adopting an edge-on orientation on the substrate, while the surrounding pink amorphous regions depict the disordered molecular arrangements. Reprinted figure with permission from ref. [39]. Copyright (2019) Springer Nature.

Chain alignment and crystallinity are particularly influential in defining anisotropic charge-transport characteristics in conjugated polymer thin films. As illustrated in Figure 5, when polymer backbones are aligned parallel to the substrate or along the charge transport direction, intrachain transport is enhanced through delocalized π-orbitals along the conjugated backbone. Concurrently, ordered interchain π–π stacking facilitates coherent charge hopping between adjacent chains and reduces energetic barriers for transport. High degrees of crystallinity give rise to extended, low-defect domains that form continuous pathways for efficient carrier migration, thereby minimizing trap-assisted recombination and improving charge diffusion. In contrast, disordered or amorphous regions disrupt π–π stacking, leading to localized states that impede charge motion and diminish overall device performance. The extent of molecular ordering and crystalline coherence can be quantitatively analyzed using techniques such as X-ray diffraction (XRD) and Grazing Incidence Wide-Angle X-ray Scattering (GIWAXS), which provide insights into lamellar spacing, π–π stacking distance, and domain orientation. These structural parameters are often found to correlate strongly with charge carrier mobility in polymer-based field-effect transistors and photovoltaic devices.

**Figure 6:**
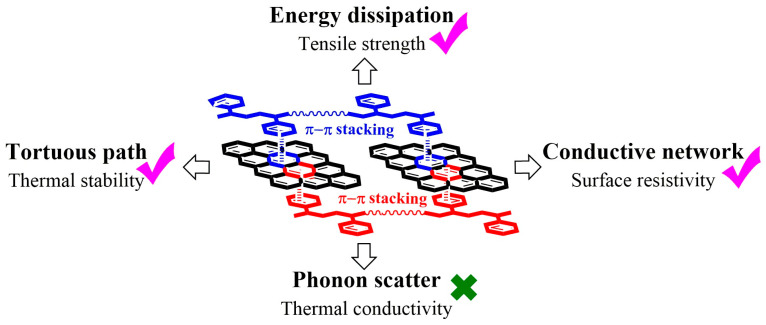
Schematic illustration of π–π stacking interactions and their multifunctional impacts on material properties. π–π stacking between conjugated backbones facilitates charge transport by forming conductive networks that lower surface resistivity, while tortuous pathways improve thermal stability. This figure is adapted from ref. [40] (reproduced under terms of the CC-BY license).

As depicted in Figure 6, π–π stacking not only underpins charge transport through the formation of conductive networks but also influences a broad spectrum of physical properties. Enhanced stacking promotes energy dissipation and mechanical robustness, while tortuous pathways between stacked domains improve thermal stability. Conversely, excessive stacking-induced phonon scattering can limit thermal conductivity. Thus, molecular design and post-deposition processing strategies—such as solvent vapor annealing, thermal annealing, or mechanical alignment—serve as effective approaches to finely tune π–π stacking and optimize the overall transport and mechanical performance of organic semiconductors.

At the molecular level, the π–π stacking distance—typically ranging from 3.3 to 3.8 Å in high-performance systems—represents a critical structural parameter governing electronic coupling between adjacent polymer chains [41]. Shorter stacking distances enhance orbital overlap and broaden electronic bandwidths, thereby promoting delocalized charge transport and reducing hopping resistance. Subtle variations in π–π stacking can arise from differences in backbone planarity, steric hindrance from side chains, or solvent-induced aggregation during film formation.

Among the molecular design factors, regioregularity exerts a profound impact on the self-assembly and crystallization behavior of conjugated polymers. High regioregularity ensures a uniform sequence of substituents along the backbone, favoring planar conformations that support tight π–π stacking and long-range order. This structural regularity minimizes torsional defects and enhances electronic delocalization. In contrast, regiorandom configurations disrupt conjugation continuity, induce chain torsion, and limit crystallite coherence length, leading to lower charge mobilities. The classic example is poly(3-hexylthiophene) (P3HT), where highly regioregular variants display mobilities one to two orders of magnitude higher than their regiorandom analogues. This correlation between molecular regularity, packing, and charge mobility is now well established across a wide range of conjugated polymer systems.

Side-chain engineering further provides a versatile molecular handle to fine-tune solubility, film morphology, and interchain interactions. Side chains govern how polymers assemble in the solid state by mediating steric and noncovalent interactions between neighboring chains. Bulky or branched side chains can inhibit close packing, reduce crystallinity, and expand π–π stacking distances, thereby limiting charge transport [42]. However, appropriately designed side chains can improve solubility during processing, promote controlled self-assembly, and enable alignment of crystalline domains with respect to the substrate. The incorporation of polar, fluorinated, or hydrogen-bonding moieties into side chains can additionally modulate aggregation kinetics, microphase separation, and dielectric environment—factors that influence both the morphology and energetics of charge transport. Recent developments in side-chain design, such as using semifluorinated chains or π-conjugated side groups, have demonstrated remarkable control over molecular orientation and interlayer coupling, leading to enhanced mobility and stability in OFETs and OPVs.

Ultimately, the interplay between chain alignment, crystallinity, regioregularity, and side-chain design defines the delicate balance between order and processability in organic semiconductors. Achieving an optimal molecular packing motif—characterized by planar backbones, dense π–π stacking, and well-aligned crystalline domains—is a prerequisite for realizing high charge carrier mobilities and reproducible device performance. Continued efforts in molecular engineering, guided by advanced structural characterization and multiscale modeling, are expected to deepen our understanding of how microscopic packing parameters dictate macroscopic electronic behavior in organic electronic materials.

#### 2.3.2. Amorphous Regions, and Grain Boundaries, and Structural Heterogeneity

Charge transport in conjugated polymers is intrinsically influenced by the coexistence of ordered and disordered regions within the solid-state microstructure. While crystalline domains facilitate efficient intrachain and interchain transport through extended π–π stacking and coherent orbital overlap, amorphous regions act as barriers that disrupt charge percolation pathways. These amorphous segments typically exhibit higher energetic disorder and localized electronic states, leading to carrier trapping and thermally activated hopping transport. The spatial distribution and connectivity of crystalline and amorphous phases therefore determine the effective charge transport dimensionality. Optimizing the degree of crystallinity and reducing structural disorder are thus essential strategies for achieving balanced mobility, mechanical flexibility, and processability in polymer semiconductors.

Grain boundaries, which represent the interfaces between misaligned crystalline domains, further modulate transport properties by introducing discontinuities in molecular orientation and packing density. At these boundaries, imperfect π–π overlap, voids, or chemical impurities can lead to localized potential barriers and trap-assisted recombination. However, not all grain boundaries are detrimental; certain semi-ordered interfacial regions can act as flexible connectors that mitigate mechanical strain and maintain electronic connectivity. Recent studies employing advanced characterization techniques such as GIWAXS, near-edge X-ray absorption fine structure (NEXAFS), and various microscopy have revealed that the degree of coherence across grain boundaries is highly dependent on molecular design and film-processing conditions.

Therefore, a comprehensive understanding of the interplay between amorphous regions, grain boundaries, and structural heterogeneity is critical for advancing next-generation polymer electronics. Strategies such as molecular engineering to promote self-assembly, use of high-boiling-point solvent additives to induce controlled phase separation, and post-deposition treatments like solvent vapor or thermal annealing have proven effective in tuning microstructural order. By carefully balancing crystalline continuity with moderate disorder, researchers can design polymer systems that achieve both high charge mobility and mechanical robustness—two key requirements for flexible and stretchable electronic applications.

### 2.4. Techniques for Probing Structure

Understanding the relationship between molecular packing, microstructure, and charge transport in polymeric semiconductors requires advanced characterization techniques capable of resolving both structural and electronic features at multiple length scales. Various experimental methods have been developed to probe chain alignment, crystallinity, π–π stacking, and morphological heterogeneity, providing critical insights into structure–property relationships. Among these, X-ray scattering, electron microscopy, and vibrational spectroscopy are particularly effective in revealing how molecular arrangement influences macroscopic device performance.

#### 2.4.1. Grazing Incidence Wide-Angle X-Ray Scattering (GIWAXS)

**Figure 7:**
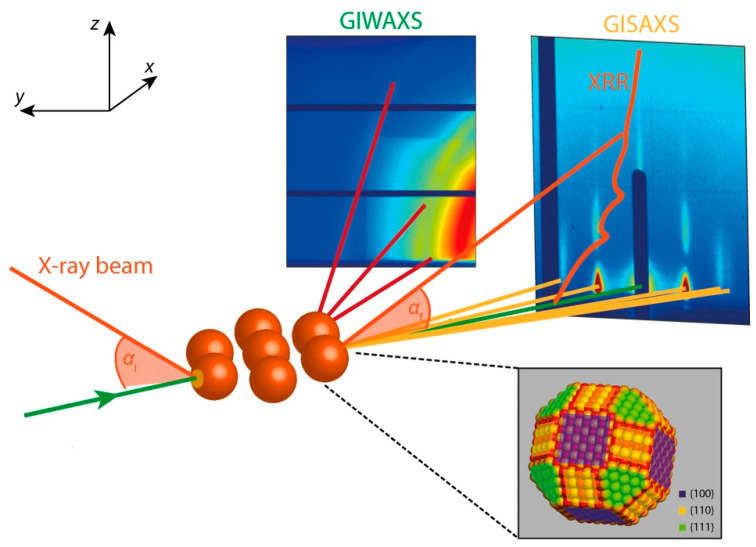
Schematic explaining the concept of the GISAXS/GIWAXS setup. Measurements are taken at a fixed incident angle relative to the liquid surface. GISAXS and GIWAXS signals are collected by detectors positioned in the forward and higher-angle directions, respectively. The inset shows a model of a nanocrystal with different facets labeled as {100}, {110}, and {111}, corresponding to diffraction features observed in the scattering patterns. The red curve in GISAXS indicates X-ray reflectivity (XRR), while the colored scattering maps depict typical intensity distributions in GIWAXS (green) and GISAXS (orange). Reprinted figure with permission from ref. [43]. Copyright (2020) Springer Nature.

GIWAXS is a powerful technique for investigating the crystalline structure and molecular orientation of thin polymer films. By directing X-rays at a shallow incident angle relative to the substrate (α), GIWAXS selectively probes near-surface regions, providing insights into π–π stacking distances, lamellar packing, and in-plane versus out-of-plane chain orientations. For example, in a P3HT:PCBM blend, GIWAXS can reveal the edge-on orientation of polymer crystallites, their layer spacing, and crystallite size, as well as the presence of fullerene aggregates (See Figure 7). Analysis of the resulting 2D scattering patterns (q_x_, q_z_) enables quantification of these structural parameters, which are directly correlated with charge transport and optoelectronic performance in devices such as organic photovoltaics and field-effect transistors.

#### 2.4.2. Scanning Electron Microscopy (SEM) and Transmission Electron Microscopy (TEM)

SEM and TEM are powerful characterization techniques that provide complementary insights into the mesoscale and nanoscale morphology of conjugated polymer thin films. SEM primarily probes the film surface, offering high-resolution images that capture topographical features, surface roughness, and phase-separated morphology arising from differences in polymer crystallinity or blend composition. For example, SEM has been used to visualize fibrillar networks in P3HT films and to monitor domain sizes in P3HT:PCBM bulk heterojunctions, revealing correlations between surface texture and device efficiency [44]. Through contrast generated by variations in secondary or backscattered electron signals, SEM can delineate crystalline versus amorphous regions and detect phase segregation, which directly influences exciton dissociation and charge transport [45].

TEM, in contrast, provides direct visualization of the internal nanostructure with sub-nanometer resolution, enabling detailed observation of lamellar stacking, π–π stacking arrangements, and crystalline domain organization [46]. Bright-field TEM, dark-field TEM, and high-resolution TEM (HRTEM) can resolve lattice fringes corresponding to lamellar spacings and π–π stacking distances, offering quantitative structural information that complements X-ray diffraction measurements [47]. Selected area electron diffraction (SAED) can further analyze crystalline orientation, domain size, and the degree of texture within the film. Practical examples include the visualization of donor–acceptor interpenetrating networks in high-performance OPVs, where TEM directly reveals the nanoscale morphology responsible for efficient charge separation. Advanced TEM techniques, including energy-filtered TEM (EFTEM) and scanning TEM (STEM) combined with electron energy loss spectroscopy (EELS), allow compositional mapping and chemical contrast at the nanometer scale. These methods are particularly valuable for multicomponent systems, such as polymer:fullerene or polymer:non-fullerene acceptor blends, providing insights into donor–acceptor phase purity, interfacial mixing, and compositional gradients [48]. Recent developments in aberration-corrected STEM, in situ TEM, and cryo-TEM have opened new possibilities for observing dynamic morphological changes, degradation mechanisms, and real-time crystallization processes under operational conditions.

Overall, SEM and TEM together provide a comprehensive morphological framework that bridges mesoscale and nanoscale regimes. By combining surface topography, internal nanostructure, and compositional mapping, these microscopy techniques offer critical insights into the formation of percolation pathways, connectivity of crystalline domains, and resultant charge transport properties in polymer semiconductors. Such detailed structural understanding guides rational material design and optimization for high-performance, next-generation polymer electronic devices.

#### 2.4.3. Raman Spectroscopy

Raman spectroscopy is a powerful, non-destructive vibrational technique that provides detailed insight into the molecular structure, conformation, and electronic delocalization of conjugated polymers. By probing the inelastic scattering of monochromatic light, Raman spectroscopy sensitively detects vibrational modes associated with specific chemical bonds and molecular backbones, thereby revealing subtle variations in conjugation length, chain planarity, and intermolecular interactions. In conjugated polymers, characteristic Raman-active modes such as the C=C stretching and C–C skeletal vibrations serve as fingerprints for the degree of π-electron delocalization and the extent of bond length alternation (BLA). Shifts in these vibrational bands are often correlated with changes in molecular ordering, doping level, and local electronic environment, allowing Raman analysis to act as a direct probe of structural and electronic coupling within the polymer framework. The technique is particularly valuable for studying chain conformational order and backbone planarity, both of which are critical determinants of charge carrier mobility and optical absorption. For instance, a redshift and narrowing of the C=C stretching mode generally indicate enhanced backbone planarity and extended conjugation, leading to improved intermolecular orbital overlap and more efficient charge transport. Conversely, blueshifts and broadening of Raman peaks suggest increased torsional disorder or localized defects, which disrupt π-conjugation and reduce electronic delocalization. Thus, Raman spectroscopy provides a molecular-level window into how structural disorder influences the macroscopic performance of polymer semiconductors.

**Figure 8:**
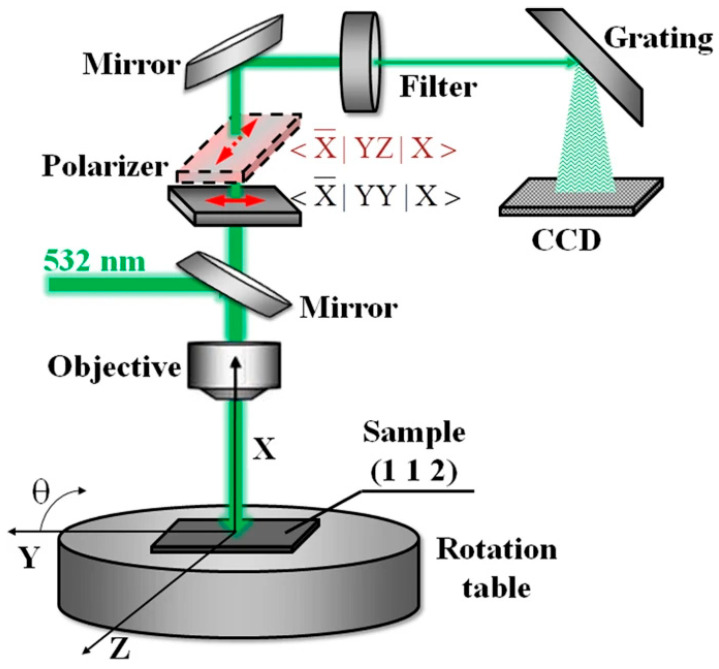
A schematic illustration depicting the detailed experimental setup employed for the polarized Raman spectroscopy measurements carried out on the (112) crystallographic plane, highlighting the orientation of the sample, polarization directions of the incident and scattered light, and the overall optical geometry used during the experiments. This figure is adapted from ref. [49] (reproduced under terms of the CC-BY license).

Polarized Raman spectroscopy further extends this capability by enabling quantitative assessment of molecular orientation and anisotropy within thin films. By varying the polarization direction of the incident and scattered light, one can determine the alignment of the polymer backbones relative to the substrate or processing direction. As illustrated in Figure 8, a 532 nm excitation beam is directed onto the sample through an objective lens, with the incident and scattered light passing through a controllable polarizer. By systematically varying the polarization directions of both the excitation and detection paths and rotating the sample about its axis (θ), one can quantitatively determine the orientation of polymer backbones relative to the substrate or processing direction. The scattered Raman signals are analyzed through a spectrometer equipped with a grating and CCD detector, enabling high-sensitivity detection of polarization-dependent vibrational modes. When combined with GIWAXS data, this approach provides a comprehensive understanding of chain alignment, crystallinity, and interchain ordering. Furthermore, the ability to perform spatially resolved Raman mapping allows visualization of heterogeneities—such as local variations in crystallinity, strain, or chemical composition—across the thin film.

Beyond structural characterization, Raman spectroscopy can also prove dynamic processes and electronic interactions. For example, it has been used to monitor polaron formation in Poly[[4,8-bis[(2-ethylhexyl)oxy]benzo[2-b:4,5-b′]dithiophene-2,6-diyl][3-fluoro-2-[(2-ethylhexyl)carbonyl]thieno[4-b]thiophenediyl]] (PTB7)-based donor and phenyl-C71-butyric acid methyl ester (PC71BM) acceptor co-polymers, tracking molecular ordering changes in PTB7:PC71BM photovoltaic blends during illumination and directly correlating these structural changes with charge transport and device performance [50]. Resonant Raman spectroscopy, where the excitation energy is tuned near the electronic transition of the polymer, amplifies specific vibrational modes coupled to the π–π* electronic transition. This resonance enhancement provides insights into exciton–phonon coupling, charge-induced structural relaxation, and polaron formation in doped or photoexcited polymers. Moreover, time-resolved or in situ Raman measurements under applied bias or illumination enable direct monitoring of structural evolution during device operation, linking molecular reorganization to electrical performance degradation or stability.

Recent advances in Raman instrumentation, such as tip-enhanced Raman spectroscopy (TERS), confocal Raman mapping, and ultrafast femtosecond Raman techniques, provide nanoscale spatial resolution and femtosecond temporal resolution, opening new possibilities for characterizing polymer domains, interfaces, and transient photophysical processes. These cutting-edge techniques allow researchers to visualize heterogeneity, monitor real-time molecular dynamics, and directly correlate structural features with device performance, thus accelerating the rational design of high-efficiency and stable polymeric semiconductor devices.

#### 2.4.4. UV–Vis Absorption

UV–Vis absorption and photoluminescence (PL) spectroscopy are indispensable optical characterization techniques that provide fundamental insights into the electronic structure, excitonic behavior, and degree of molecular order in conjugated polymers. These techniques probe the interactions between light and the delocalized π-electron system, thereby offering a direct connection between molecular structure and optoelectronic functionality. Together, they reveal key information on optical bandgaps, conjugation length, aggregation state, and exciton dynamics, all of which are central to understanding charge generation and transport in organic electronic materials. UV–Vis absorption spectroscopy measures the electronic transitions from the ground to excited states within the conjugated backbone, reflecting the extent of π–π* interactions and conjugation length. In conjugated polymers, the absorption spectrum is typically composed of a broad π–π* transition and vibronic progressions associated with backbone planarity and intermolecular coupling. The onset of the absorption spectrum defines the optical bandgap (*E*_g_), which serves as a critical parameter for determining energy level alignment in devices such as OPVs and OFETs.

Variations in absorption peak position and shape provide valuable information on molecular packing and chain conformation. A redshift in the maximum absorption and the emergence of distinct vibronic peaks indicate enhanced interchain coupling, planarization of the polymer backbone, and increased effective conjugation length—features often associated with improved charge transport and photocarrier generation efficiency. Conversely, blueshifts or broadened spectra are indicative of structural disorder, torsional twisting, or reduced conjugation, which localize excitons and hinder electronic communication between neighboring chains.

#### 2.4.5. PL Spectroscopy

PL spectroscopy provides complementary insights by probing the radiative recombination of photoexcited species. The PL spectrum reflects the energy distribution of excitons and the degree of electronic coupling between polymer chains. The spectral shape, Stokes shift, and vibronic structure of PL emissions serve as sensitive indicators of exciton delocalization, chain conformation, and aggregation type. For example, a smaller Stokes shift and distinct vibronic progression suggest a more ordered and planarized molecular arrangement with reduced structural relaxation upon excitation. The comparison between absorption and PL spectra offers valuable information on exciton dynamics. The overlap between the two spectra can be analyzed using the Förster resonance energy transfer (FRET) framework to quantify energy transfer efficiency within blended or multilayer systems. In conjugated polymer blends, PL quenching is often used to evaluate exciton dissociation efficiency and donor–acceptor interfacial interactions. A significant quenching of PL intensity in donor–acceptor blends indicate efficient charge transfer and exciton separation—an essential mechanism for achieving high power conversion efficiency in organic solar cells.

Time-resolved photoluminescence (TRPL) measurements further complement steady-state spectroscopy by providing dynamic insights into the fundamental processes governing excited-state behavior. Through monitoring the temporal evolution of the PL decay, TRPL enables quantitative evaluation of exciton lifetimes, recombination dynamics, and non-radiative decay pathways. Shortened PL lifetimes are typically associated with efficient exciton dissociation or rapid charge transfer at donor–acceptor interfaces, reflecting favorable pathways for charge separation. In contrast, prolonged lifetimes may indicate the presence of isolated or energetically trapped domains with limited interfacial contact, where excitons undergo radiative recombination rather than contributing to charge generation. These dynamic observations are particularly valuable for elucidating the influence of molecular packing, energetic disorder, and trap states on charge carrier dynamics. By correlating the TRPL-derived kinetic parameters with device metrics such as photocurrent response or power conversion efficiency, one can establish a direct link between the photophysical properties of conjugated polymers and their macroscopic optoelectronic performance.

#### 2.4.6. Atomic Force Microscopy (AFM)

AFM is one of the most powerful and versatile techniques for probing nanoscale morphology, surface roughness, and mechanical properties of conjugated polymer thin films. By scanning a sharp tip across the sample surface and monitoring the tip–sample interactions, AFM provides three-dimensional topographical information with sub-nanometer vertical resolution. Its ability to image non-conductive materials under ambient or controlled environments makes it particularly suitable for the characterization of polymer semiconductors, organic electronic devices, and nanostructured thin films.

In its most common operation mode—tapping or intermittent contact—AFM enables detailed visualization of film morphology, revealing nanoscale features such as fibrillar aggregates, crystalline domains, and amorphous regions. These morphological signatures directly reflect the self-assembly behavior of conjugated polymer chains during film formation. For example, semicrystalline polymers such as poly(3-hexylthiophene) (P3HT) typically exhibit nanofibrillar textures arising from π–π stacking and lamellar organization, whereas donor–acceptor copolymers often form interconnected domain networks that govern charge percolation pathways. Quantitative analysis of AFM height images provides key metrics such as root-mean-square (RMS) roughness, grain size, and surface coverage, which serve as indicators of the film’s structural uniformity and crystallinity. Lower surface roughness typically correlates with improved thin-film continuity and reduced interfacial charge traps, beneficial for device performance in field-effect transistors (FETs) and solar cells. Moreover, image analysis using fast Fourier transform (FFT) or height–height correlation functions allow researchers to assess the periodicity and orientation of nanostructures, offering deeper insight into anisotropic chain alignment induced by solution shearing, blade coating, or zone casting.

## 3. Synthesis and Processing Techniques

The performance of polymeric semiconductors is governed not only by their intrinsic electronic properties but also by the methods used for synthesis and film formation. Molecular design defines the backbone planarity, conjugation length, and functional group incorporation, which determine the optoelectronic characteristics such as bandgap, HOMO–LUMO alignment, and charge mobility. Simultaneously, processing techniques, including thin-film deposition, patterning, and interface engineering, dictate the mesoscale morphology, chain alignment, and crystallinity of the active layer, all of which are crucial for device efficiency, operational stability, and reproducibility. Recent advances in scalable and environmentally friendly synthesis, precise film engineering, and interface optimization have enabled polymeric semiconductors to achieve performance levels approaching those of inorganic counterparts, expanding their applicability in next-generation electronics.

### 3.1. Synthetic Approaches

The design of conjugated polymer backbones relies on synthetic strategies that allow precise control over regioregularity, molecular weight, and functionalization. Achieving well-defined polymers is critical, as minor defects or irregularities in the chain can lead to energetic disorder, trap formation, and reduced charge transport. Synthetic approaches can be broadly divided into step-growth cross-coupling reactions, post-polymerization modifications, and emerging green and scalable methodologies. Each strategy provides different advantages in terms of structural control, functional group incorporation, and compatibility with sustainable production.

#### 3.1.1. Coupling Strategies for Conjugated Polymer Synthesis

##### Direct Arylation Polymerization of Thiophene Derivatives

**Figure 9:**
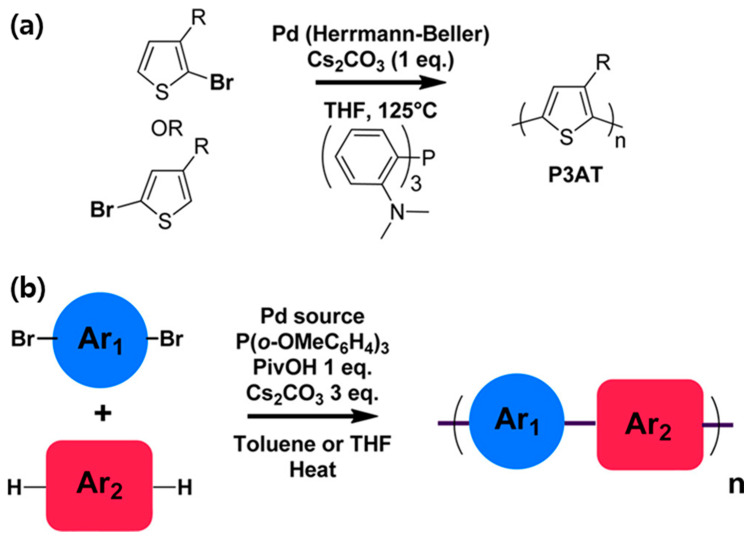
General polymerization strategies for aromatic monomers: (**a**) homopolymerization of alkyl-substituted thiophenes to P3ATs, and (**b**) cross-coupling of bromoarenes (Ar_1_) with heterocycles (Ar_2_) to produce diverse conjugated copolymers, enabling well-defined polymers with properties comparable or superior to traditional methods. Reprinted figure with permission from ref. [51]. Copyright (2016) American Chemical Society.

Panel (a) in Figure 9 depicts the polymerization of substituted thiophene monomers to form poly(3-alkylthiophene) (P3AT), a widely studied class of conjugated polymers with applications in organic photovoltaics, field-effect transistors, and light-emitting devices. The reaction is catalyzed by a palladium complex, commonly the Herrmann–Beller type, in the presence of cesium carbonate (Cs_2_CO_3_) as a base and tetrahydrofuran (THF) as the solvent at elevated temperatures (~125 °C). Mechanistically, the Pd catalyst undergoes oxidative addition with the brominated thiophene monomer, forming a Pd (II) intermediate. Subsequent transmetalation and reductive elimination steps lead to the formation of C–C bonds between monomer units, propagating the polymer chain. The substituent R on the thiophene ring, typically an alkyl or alkoxy group, modulates solubility, molecular weight, and the degree of order in the solid state. The resulting P3AT exhibits high π-conjugation along the polymer backbone, with the regioregularity of the coupling dictating the crystallinity, charge transport, and optical properties of the polymer.

##### Suzuki–Miyaura Polycondensation for Donor–Acceptor Polymers

Panel (b) in Figure 9 illustrates a more general strategy for synthesizing donor–acceptor (D–A) conjugated polymers via Suzuki–Miyaura polycondensation. In this approach, a dibromo-substituted aromatic monomer (Ar_1_) is coupled with a bis-boronic acid (or boronate ester)-substituted aromatic monomer (Ar_2_) in the presence of a Pd catalyst. The reaction employs a phosphine ligand, typically tris(ortho-methoxyphenyl)phosphine, and pivalic acid (PivOH) as an additive to enhance catalytic efficiency, along with Cs_2_CO_3_ as a base. The reaction is conducted in toluene or THF under heating conditions, enabling the formation of alternating Ar_1_–Ar_2_ repeat units along the polymer chain. This D–A polymerization strategy provides precise control over the electronic properties of the resulting polymer, as the donor and acceptor moieties dictate the HOMO–LUMO energy levels, optical bandgap, and charge-carrier mobility. By carefully selecting monomer structures, chemists can fine-tune polymer solubility, molecular weight distribution, and backbone planarity, which are critical parameters for device performance.

#### 3.1.2. Post-Polymerization Modifications

**Figure 10:**
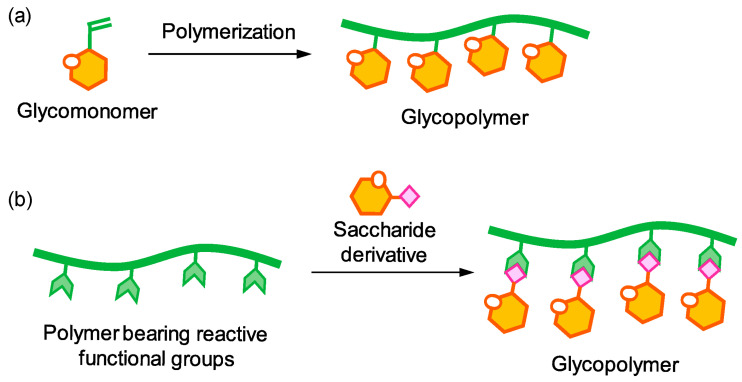
Synthetic routes for glycopolymers via (**a**) direct polymerization of glycomonomers and (**b**) post-polymerization modification of pre-formed polymers. This figure is adapted from ref. [52] (reproduced under terms of the CC-BY license).

Figure 10 illustrates the strategy of post-polymerization modification, a powerful approach for tailoring polymer properties without compromising the integrity of the conjugated backbone. In this approach, polymers bearing chemospecific handles introduced during controlled polymerization react with various bifunctional reagents to achieve precise functionalization. Post-polymerization modifications include reactions with active esters, anhydrides, isocyanates, oxazolones, and epoxides, as well as Michael-type additions, thiol exchange, radical thiol or atom transfer radical additions, reactions with aldehydes and ketones, Huisgen 1,3-dipolar cycloadditions, and Pd-catalyzed (cross-)coupling reactions. These chemical transformations allow systematic tuning of side chains and end groups to modulate solubility, film formation, self-assembly, and energy levels. For example, incorporation of alkyl or oligoethylene glycol side chains enhances solubility and promotes ordered microstructures, while polar or electron-rich/deficient functional groups can optimize interfacial energy-level alignment and charge injection. Moreover, post-polymerization cross-linking stabilizes thin-film morphology, preventing dewetting or undesired crystallite reorganization during thermal or solvent annealing. Collectively, these strategies provide a versatile toolbox for designing functional polymers with properties tailored to specific optoelectronic device applications.

#### 3.1.3. Green and Scalable Synthesis Methods

With growing emphasis on sustainability and industrial feasibility, green and scalable polymerization methods are increasingly important. Solvent-free polymerizations, aqueous-phase reactions, and metal-free catalytic systems minimize environmental impact while maintaining high polymer quality. Furthermore, strategies that reduce energy consumption and hazardous byproducts are critical for large-scale production. For example, microwave-assisted polymerization and continuous-flow reactors allow fast, uniform, and scalable polymer synthesis. Integration of these methods with high-throughput purification and characterization techniques ensures that industrially relevant polymers retain the optoelectronic performance observed in laboratory-scale studies. By addressing sustainability and scalability, these approaches bridge the gap between fundamental research and commercial application of polymeric semiconductors.

### 3.2. Thin-Film Processing and Patterning

The optoelectronic properties of polymeric semiconductors in devices depend strongly on the mesoscale organization achieved during thin-film processing. Polymer chains self-assemble into crystalline and amorphous domains, with the degree of order and orientation directly affecting charge transport pathways. Thin-film morphology is influenced by deposition method, solvent choice, drying kinetics, substrate interactions, and thermal or solvent annealing. Optimization of these parameters enables uniform films with controlled thickness, domain connectivity, and minimal defect density, which are essential for achieving high mobility and device stability.

#### 3.2.1. Solution Processing (Spin-Coating, Inkjet, Blade Coating)

Solution-based deposition methods are widely used for fabricating large-area polymer films. Spin-coating offers rapid, uniform coverage for laboratory-scale devices, while inkjet and blade coating enable patterned deposition suitable for scalable and flexible electronics. The choice of method affects film thickness, uniformity, and solvent evaporation rate, which in turn influence crystallinity and molecular orientation. Blade coating and slot-die techniques are particularly compatible with roll-to-roll processing, allowing industrial-scale fabrication of organic photovoltaics, transistors, and light-emitting devices. Control over deposition parameters, such as coating speed, substrate temperature, and solution concentration, is critical for producing reproducible and high-performance films.

#### 3.2.2. Alignment Control and Film Uniformity

Molecular alignment within the thin film is crucial for efficient charge transport. Techniques such as zone-casting, meniscus-guided coating, and shear-assisted deposition induce preferential orientation of polymer backbones, enhancing intrachain π–π overlap and charge delocalization. Edge-on or face-on orientations relative to the substrate can be tailored to optimize either lateral or vertical charge transport depending on device requirements. Uniform film thickness and minimized surface defects reduce scattering sites and trap states, ensuring stable electrical performance. The combination of alignment control with post-deposition annealing allows tuning of crystalline domain size and connectivity, further improving carrier mobility and device reproducibility.

#### 3.2.3. Additive and Solvent Engineering

Solvent choice, cosolvent systems, and processing additives play a central role in controlling polymer aggregation and film morphology. High-boiling point solvents or selective cosolvents can slow evaporation, promoting larger crystalline domains and improved π–π stacking. Additives such as small-molecule surfactants or nucleating agents can enhance crystallinity, reduce domain boundaries, and improve film uniformity. Solvent engineering is particularly important for donor–acceptor polymers, where subtle changes in solvent polarity or evaporation rate can dramatically alter phase separation and microstructure. Careful optimization of these parameters enables a balance between solubility, self-assembly, and charge transport efficiency.

### 3.3. Doping and Interface Engineering

Doping and interfacial engineering are critical strategies for modulating carrier density, energy-level alignment, and charge injection/extraction efficiency in polymeric semiconductors. Proper selection of dopants and interlayers enables control over electronic properties while minimizing trap formation and degradation under operational conditions. The combined optimization of bulk doping and interfacial layers can significantly improve device performance, especially in field-effect transistors, light-emitting diodes, and neuromorphic devices.

#### 3.3.1. Doping and Dopants

**Figure 11:**
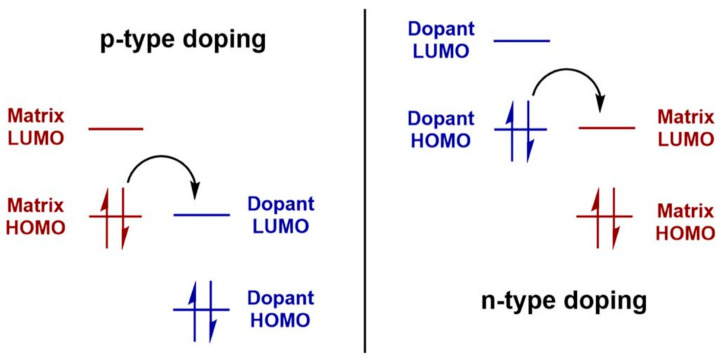
Doping in organic semiconductors can be understood through the integer charge transfer model, which describes the process as a complete electron transfer between the dopant and the semiconductor molecule. The arrows indicate the direction of complete electron transfer between matrix and dopant. In p-type doping, electrons move from the matrix HOMO to the dopant LUMO, generating holes in the matrix. In n-type doping, electrons transfer from the dopant HOMO to the matrix LUMO, producing free electrons in the matrix. This figure is adapted from ref. [53] (reproduced under terms of the CC-BY license).

Chemical or molecular doping is a critical strategy for modulating the electronic properties of conjugated polymers (CPs). As depicted in Figure 11, doping can be classified into n-type and p-type processes. In n-type doping, electrons are added to the polymer backbone, thereby increasing the density of negative charge carriers. Conversely, p-type doping introduces electron holes, creating positive charge carriers that facilitate hole transport. This selective addition of charge carriers directly influences the polymer’s conductivity, charge mobility, and overall electronic performance. The choice of dopants plays a decisive role in the efficiency and stability of the doping process. As illustrated in the Figure 11, a wide variety of dopants are employed: neutral species such as halogens (Br_2_, I_2_), ionic salts (FeClO_4_, LiClO_4_), organic acids (CH_3_COOH, CF_3_SO_3_Na), polymeric dopants (PVA, PVS), and metal oxides (SnO_2_, TiO_2_). Each dopant class interacts with the conjugated polymer in unique ways, influencing the charge transfer efficiency, polymer morphology, and long-term chemical stability.

Effective doping not only enhances charge carrier density but also improves charge injections and reduces contact resistance at electrode interfaces. It can further mitigate energetic disorder by stabilizing the polymer’s electronic structure. Achieving uniform dopant distribution is particularly important in high-performance devices, such as OFETs and OPVs, where spatial inhomogeneity can create trap states that impede charge transport. Controlled doping strategies, therefore, enable precise tuning of electrical properties without disrupting the polymer’s microstructure, ensuring reliable device operation over extended periods.

#### 3.3.2. Energy-Level Tuning and Interfacial Layers

Energy-level alignment is a critical factor governing the efficiency of charge injection and extraction in polymeric semiconductors. Interfacial engineering strategies—such as the introduction of hole-transport layers (HTLs), electron-transport layers (ETLs), self-assembled monolayers (SAMs), or buffer layers—allow precise tuning of the energetic landscape at the polymer–electrode interface. By minimizing the mismatch between the polymer’s highest occupied molecular orbital (HOMO) or lowest unoccupied molecular orbital (LUMO) and the electrode work function, these interlayers reduce injection barriers, facilitate more efficient charge collection, and suppress unwanted charge recombination.

Beyond simple energy-level matching, interfacial layers can influence polymer morphology at the nanoscale. For instance, certain SAMs induce preferential molecular orientation, improving π–π stacking and in-plane charge transport. Similarly, buffer layers can modulate interfacial dipoles, which adjust local electric fields and promote uniform charge injections across the active layer. Functional interlayers also serve to passivate surface traps that would otherwise act as recombination centers, enhancing operational stability under continuous electrical bias. Combining controlled doping with such interfacial engineering strategies enables balanced hole and electron transport, mitigates space-charge accumulation, and significantly improves device power conversion efficiency and operational lifetime.

Recent advances in molecular design have further expanded the scope of interfacial engineering. For example, conjugated polyelectrolytes and dipolar interlayers have been used to create tunable interfacial energy offsets, enabling the selective enhancement of either electron or hole injection. Additionally, the introduction of ultrathin, cross-linkable interlayers can maintain interfacial integrity under thermal and mechanical stress, which is essential for flexible or wearable devices. Collectively, these approaches highlight the interplay between molecular design, energy-level engineering, and interfacial chemistry in achieving high-performance polymer electronic devices.

#### 3.3.3. Stability and Environmental Effects

Polymer semiconductors are inherently sensitive to environmental factors such as oxygen, moisture, and temperature, which can profoundly affect their electronic and structural properties. Exposure to oxygen or water often leads to oxidative doping, trap formation, or chain scission, resulting in reduced carrier mobility and increased recombination losses. Thermal stress can induce morphological changes, such as polymer chain rearrangement or phase segregation, which alter energy levels and disrupt charge transport pathways.

To mitigate these effects, encapsulation strategies—including thin-film barriers, multilayer coatings, or inert atmosphere packaging—are frequently employed. Additionally, careful selection of dopants that are thermally and chemically stable, combined with interfacial engineering, can prevent unintended reactions at the polymer–electrode interface. Understanding the interactions between polymer chains, dopants, and environmental species allows for the rational design of devices with extended lifetimes, minimal hysteresis, and reproducible performance under variable operating conditions.

Advanced stabilization techniques have further expanded the toolkit for enhancing environmental resilience. Cross-linkable side chains or backbone modifications can reduce polymer chain mobility, limiting morphological degradation under heat or bias stress. Protective interlayers—such as metal oxides or fluorinated polymers—act as both physical barriers and electronic passivation layers, improving resistance to oxidative and moisture-induced degradation. Furthermore, strategies that combine chemical stabilization with morphological control, such as the incorporation of rigid rod-like segments or crystalline domains, have demonstrated substantial improvements in long-term device stability. These insights underscore the importance of integrating chemical, morphological, and interfacial approaches to engineering polymer semiconductors that are both high-performing and durable.

## 4. Applications in Next-Generation Electronics

Organic semiconductors, owing to their tunable electronic energy levels, solution processability, and inherent mechanical flexibility, have emerged as critical materials for next-generation electronic devices. Their versatile molecular structures allow precise control over optoelectronic properties, enabling the design of devices with tailored performance characteristics.

In OPVs, the efficiency of light-to-electricity conversion is heavily influenced by molecular packing, donor–acceptor energy level alignment, and interfacial engineering. Strategies such as controlled crystallinity, p-type and n-type doping, and the incorporation of hole/electron transport layers or self-assembled monolayers facilitate efficient exciton dissociation and charge extraction, minimizing recombination losses. Such optimizations have been shown to significantly enhance power conversion efficiencies while maintaining long-term device stability under operational conditions. For OFETs, the alignment of polymer chains and small-molecule semiconductors critically affects charge carrier mobility. Interfacial layers, dielectric engineering, and energy-level tuning are employed to optimize threshold voltage, reduce contact resistance, and mitigate bias stress-induced degradation. These approaches improve not only the electrical performance but also the operational reliability of OFETs, enabling their integration into flexible and large-area electronics. Emerging neuromorphic devices leverage the coupled ionic-electronic transport in conjugated polymers to mimic biological synaptic functions. By exploiting the electrochemical gating of polymer channels or the formation of ion-trapping layers, these devices can emulate short-term and long-term plasticity, spike-timing-dependent plasticity (STDP), and other complex learning rules. This opens pathways toward low-power, flexible, and bio-inspired computing architectures, including wearable and implantable systems.

Beyond OPVs and OFETs, the field of organic electronics has recently expanded into emerging applications such as neuromorphic and memristor devices. Neuromorphic devices leverage the intrinsic plasticity and tunable conductivity of organic materials to emulate synaptic functions, offering a promising route toward low-power and flexible artificial neural networks. Similarly, organic and hybrid organic–inorganic memristors exploit redox-active polymers, ionic migration, or charge-trapping mechanisms to enable non-volatile information storage and analog signal processing. These developments highlight the remarkable versatility of organic semiconductors in bridging electronic, ionic, and photonic functionalities. To achieve reliable and scalable device operation, integrated strategies involving chemical doping, interfacial engineering, encapsulation, and environmental stabilization are indispensable. In this section, we provide a comprehensive overview of these device architectures, emphasizing the underlying material design principles, charge transport and switching mechanisms, and the latest approaches to address limitations in efficiency, stability, and manufacturability. Particular attention is devoted to the synergistic interplay between molecular engineering, processing control, and device-level optimization that continues to drive organic electronics toward practical neuromorphic and memory applications.

### 4.1. Organic Photovoltaics (OPVs)

OPVs are a class of solar cells that utilize conjugated polymers or small molecules as the photoactive layer to convert sunlight into electricity. Their tunable energy levels, solution processability, lightweight nature, and mechanical flexibility make them promising candidates for next-generation, flexible, and wearable solar energy technologies. Unlike traditional silicon-based photovoltaics, OPVs rely on exciton generation, diffusion, and dissociation at donor–acceptor interfaces, followed by charge transport and extraction to electrodes. The performance of OPVs is critically dependent on molecular design, film morphology, energy-level alignment, and interfacial engineering [54].

#### 4.1.1. Device Structure and Function

**Figure 12:**
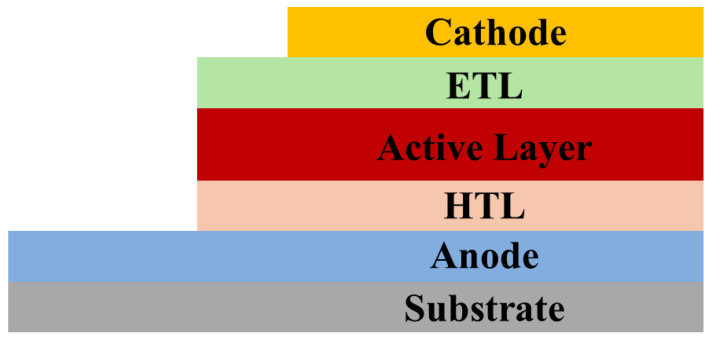
The structure of an organic solar cell comprises multiple layers, including a photoactive layer made of organic materials that absorb light and generate charge carriers, charge transport layers that facilitate the movement of electrons and holes, and electrodes that collect the charges to produce electrical current.

A typical OPV device consists of a multilayer architecture with the following configuration (Figure 12):

1. Anode Coated on Transparent Substrate: Often glass or flexible polymer coated with Indium Tin Oxide (ITO), which acts as the anode.
2. Hole Transport Layer (HTL): Materials such as PEDOT:PSS facilitate hole extraction and smoothen the anode surface.
3. Active Layer: A blend of donor (D) and acceptor (A) materials, forming a bulk heterojunction (BHJ) that provides a large interface for exciton dissociation.
4. Electron Transport Layer (ETL): Layers like ZnO or PFN optimize electron collection at the cathode.
5. Cathode: Low-work-function metals such as Al or Ag enable efficient electron extraction.

When photons are absorbed in the active layer, excitons (bound electron–hole pairs) are generated. These excitons diffuse to the D–A interface, where charge separation occurs due to the energy-level offset between donor HOMO/LUMO and acceptor HOMO/LUMO levels. Separated charges then migrate through their respective transport layers to electrodes, producing photocurrent.

The charge generation can be represented as:

(1) $$Exciton\;dissociation\overset{∆E_{DA}}{\to }e_{acceptor}^{-}+h_{donor}^{+}$$

where $\Delta E_{DA}$ is the energy offset between the donor LUMO and acceptor LUMO, which must exceed the exciton binding energy for efficient charge separation.

#### 4.1.2. Performance Metrics and Mobility Trends

OPV performance is commonly evaluated using power conversion efficiency (PCE) [55]:

(2) $$\mathrm{PCE}=\frac{J_{SC}\times V_{OC}\times FF}{P_{in}}$$

where, $J_{SC}$ = short-circuit current density (mA/cm^2^), $V_{OC}$ = open-circuit voltage (V), FF = fill factor (unitless), $P_{in}$ = incident light power (mW/cm^2^). Charge carrier mobility (μ) in the donor and acceptor phases strongly influences $J_{SC}$ and FF. For BHJ OPVs, balanced electron ($\mu_{e}$) and hole ($\mu_{h}$) mobilities reduce space-charge effects and recombination losses. The Langevin recombination rate is expressed as [56]:

(3) $$R=\gamma k_{n}np$$

where γ is Langevin reduction factor ($\gamma =\frac{k_{r}}{k_{r}+k_{s}}$), *k_s_* is the average rate constant at which CT states recombine, *k_s_* is the rate constant at which CT states split back into free carriers, n and p are electron and hole concentrations, respectively. High-performance OPVs typically exhibit μ values in the range of $10^{-4}–10^{-2}$ cm^2^/V·s for both carrier types [57].

#### 4.1.3. Tandem Photovoltaics

**Figure 13:**
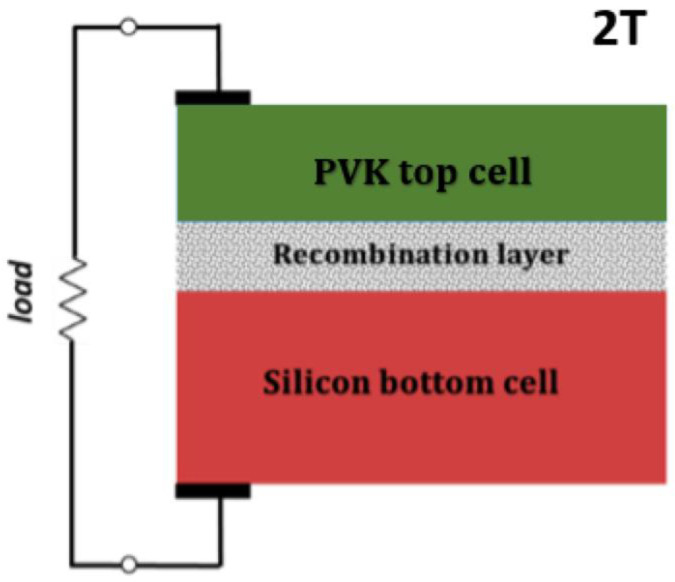
Architecture of a two-terminal tandem photovoltaic cell. Architecture of a two-terminal tandem cell. The front and rear sub-cells are composed of perovskite (PVK, wide-bandgap) and silicon (narrow-bandgap) materials, respectively. Front and rear cells are connected via a recombination layer and selective contacts, with bottom electrode and a top electrode for charge collection. This figure is adapted from ref. [58] (reproduced under terms of the CC-BY license).

Tandem OPV cells are designed to overcome the intrinsic efficiency limits of single-junction devices by stacking multiple photoactive sub-cells with complementary absorption characteristics. As shown in Figure 13 [58], a two-terminal (monolithic) tandem OPV typically consists of a wide-bandgap front sub-cell and a narrow-bandgap rear sub-cell. These sub-cells are connected by a re-combination or interconnecting layer (ICL) that enables charge recombination and optical coupling between the two photoactive layers [59,60]. 

The operating principle of tandem OPVs is based on the spectral splitting of sunlight, where the front sub-cell absorbs high-energy (short-wavelength) photons, while the rear sub-cell harvests the transmitted low-energy (long-wavelength) photons. This configuration reduces thermalization losses and unabsorbed photon losses, effectively increasing the total photocurrent and voltage output. The theoretical power conversion efficiency (PCE) of an optimized two-junction tandem cell can exceed 20–25% under standard AM1.5G illumination, compared with ~12–15% typical for single-junction OPVs [61,62].

In a monolithic tandem structure, the two sub-cells are connected electrically in series, and thus must satisfy the current-matching condition, as the total current is limited by the sub-cell with the lower photocurrent:

(4) $$J_{SC,\mathrm{tandem}}=min(J_{SC,1},J_{SC,2})$$

(5) $$V_{OC,\mathrm{tandem}}\approx V_{OC,1}+V_{OC,2}$$

To achieve efficient operation, precise control of the optical and electrical balance between the two sub-cells is essential. Optical modeling (e.g., using the transfer-matrix method) is commonly employed to determine the optimal thickness of each active layer, ensuring current matching and minimal reflection or parasitic absorption [63]. The interconnecting layer (ICL) must exhibit high optical transparency, efficient charge recombination, and chemical compatibility with both adjacent sub-cells. Typical ICL designs employ combinations of transition metal oxides (e.g., MoO_3_, V_2_O_5_, or WO_3_) and n-type materials (e.g., ZnO, TiO_X_, PFN-Br) to create ohmic contact and minimize energy barriers [64,65,66].

In addition to material engineering, energy-level alignment between donor and acceptor components within each sub-cell plays a crucial role in determining both the short-circuit current and open-circuit voltage. The voltage gain in tandem structures approximately equals the sum of the individual sub-cell voltages, thus allowing higher total V_OC_ values than in single-junction OPVs. Moreover, careful morphological control through solvent additives, thermal annealing, or sequential deposition can optimize phase separation and charge transport, further enhancing the fill factor and overall PCE [67,68].

### 4.2. Organic Field-Effect Transistors (OFETs)

**Figure 14:**
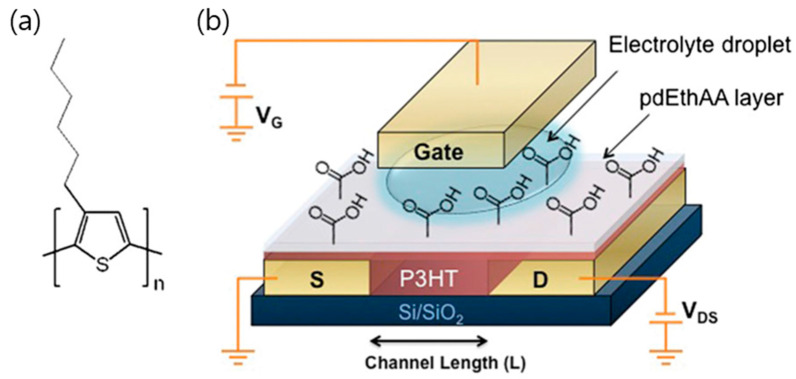
Schematic illustration of an organic field-effect transistor. (**a**) Chemical structure of P3HT, employed as the organic semiconductor. (**b**) Illustration of an electrolyte-gated organic field-effect transistor, where a plasma deposited ethylene/acrylic acid (pdEthAA) film produced via PECVD is coated on the P3HT layer. This figure is adapted from ref. [69] (reproduced under terms of the CC-BY license).

OFETs are key components in next-generation flexible and low-cost electronics, enabling applications in logic circuits, sensors, memory devices, and neuromorphic systems. A schematic of an organic field-effect transistor (OFET) is shown in Figure 14, illustrating the layered device structure with source/drain electrodes, dielectric layer, semiconductor, and a self-assembled monolayer (OTS) on a doped Si substrate, along with a molecular-level depiction of the semiconductor–surface interaction. 

Unlike inorganic FETs based on silicon or metal oxides, OFETs use semiconducting organic materials—either small molecules or conjugated polymers—as their active layers. Their charge transport occurs primarily through π–π interactions along and between polymer chains or molecular crystals. The ability to chemically tune energy levels, molecular packing, and interfacial interactions makes OFETs an attractive platform for achieving both high mobility and mechanical flexibility [70,71].

In an OFET, the drain current (*I*_D_) is modulated by an applied gate voltage (*V*_G_) that controls the charge density in the channel formed at the semiconductor–dielectric interface. The fundamental operation follows standard field-effect transistor physics, where the drain current in the saturation regime is given by ref. [72]:

(6) $$I_{D}=\frac{W}{2L}\mu C_{i}(V_{G}-V_{th}{)}^{2}$$

and in the linear regime:

(7) $$I_{D}=\frac{W}{L}\mu C_{i}\left[\left(V_{G}-V_{th}\right)V_{D}-\frac{V_{D}^{2}}{2}\right]$$

where W and L are the channel width and length, $C_{i}$ is the gate dielectric capacitance per unit area, μ is the field-effect mobility, and $V_{th}$ is the threshold voltage. These parameters define the transistor’s electrical characteristics and are crucial in evaluating the semiconductor’s intrinsic charge transport capability and interface quality.

#### 4.2.1. Active-Layer Design and Performance Metrics

The performance of OFETs is strongly dictated by the design of the organic semiconductor layer. Conjugated polymers are particularly advantageous because their molecular structure allows solution processability and mechanical compliance, essential for flexible electronics. The active layer must provide efficient charge injection, high carrier mobility, and structural stability under electrical stress. For p-type OFETs, donor–acceptor (D–A) copolymers such as PBTTT, DPP-based polymers, and derivatives have been widely studied due to their strong intramolecular charge transfer and planar backbones that promote π–π stacking. Conversely, n-type OFETs have historically lagged due to the high electron affinity required for stable electron transport. However, the development of polymeric acceptors such as NDI-, PDI-, and BBI-based systems, as well as fluorinated backbones, has enabled air-stable n-channel conduction with mobilities exceeding 1 cm^2^ V^−1^ s^−1^ [73].

At the molecular level, the charge transport in OFETs can be described by hopping or band-like mechanisms, depending on the degree of molecular order. field effect mobility as function of gate voltage in organic and inorganic amorphous TFTs has been well modeled through the expression [72]:

(8) $$\mu_{FET}=\mu_{0}\;{\exp\left(-\frac{T_{0}}{T}\right)}^{1/\alpha }$$

where $\mu_{0}$ is mobility at zero field, *T*_0_ is temperature at zero field, *T* is temperature, and α is integer ranging from 1 to 4. In highly ordered crystalline polymers, band-like transport can occur, leading to a decrease in mobility with increasing temperature, characteristic of delocalized charge carriers. Recent advances in molecular engineering, such as side-chain optimization, regioregularity control, and halogenation, have allowed fine-tuning of energy levels and improved backbone planarity. Furthermore, the introduction of self-assembled monolayers (SAMs) at the dielectric interface enhances molecular ordering and reduces interfacial traps, leading to improved mobility and reduced hysteresis.

The field-effect mobility ($\mu_{FET}$) serves as a key metric to evaluate OFET performance. Over the past decade, polymer-based OFETs have achieved mobilities rivaling amorphous silicon (≈1 cm^2^ V^−1^ s^−1^) and, in some cases, approaching polycrystalline silicon (>10 cm^2^ V^−1^ s^−1^). These advances stem from improved backbone coplanarity, strong π–π stacking (with interplanar distances of 3.4–3.6 Å), and optimized film morphology [74].

#### 4.2.2. Stability and Operational Reliability

Operational stability remains one of the most significant barriers to commercialization of OFETs. Charge trapping, interfacial polarization, and environmental degradation (oxygen and moisture uptake) lead to threshold voltage shifts, hysteresis, and mobility decay over time. These degradation processes are often quantified by bias-stress stability tests, where the time-dependent change in drain current under constant bias follows a stretched exponential model [75]:

(9) $$\Delta I_{D}(t)=I_{D}\left(0\right)\exp\left[-{\left(\frac{t}{\tau }\right)}^{\beta }\right]$$

where τ is the characteristic relaxation time and β is a dispersion parameter (0<β<1).

Improving stability involves multiple design strategies: (1) introducing encapsulation layers or hydrophobic dielectrics (e.g., CYTOP, fluorinated polymers) to suppress water and oxygen ingress; (2) molecular design of semiconductors with low-lying LUMO levels (<−4.0 eV) for n-type stability; and (3) incorporation of cross-linkable polymers to improve morphological robustness under bias and thermal stress [76,77].

#### 4.2.3. Emerging Applications and Next-Generation Directions of OFETs

Organic field-effect transistors (OFETs) have rapidly evolved beyond their initial role as flexible and low-cost electronic switches, emerging as a versatile platform for next-generation electronics. Recent advances in high-mobility organic semiconductors, interface engineering, and dielectric materials have enabled OFETs to approach performance metrics comparable to amorphous silicon, opening opportunities for high-speed logic circuits, large-area sensors, and flexible displays. Notably, molecular design strategies that enhance π–π stacking and reduce energetic disorder are now enabling devices with mobilities exceeding 10 cm^2^/V·s, a threshold that was previously unattainable for solution-processed organics [78,79].

In cutting-edge applications, OFETs are increasingly being integrated into bioelectronics and neuromorphic systems. The ability to transduce chemical or ionic signals directly into electrical responses makes OFETs ideal candidates for implantable sensors, synaptic mimics, and neuromorphic circuits. For example, electrolyte-gated OFETs have demonstrated low-voltage operation and high sensitivity in biomolecule detection, bridging the gap between conventional electronics and biological interfaces. Moreover, recent work on vertical and ambipolar OFET architectures is expanding their functional versatility, enabling complementary logic circuits and novel memory devices.

Looking forward, the field is moving toward multifunctional, hybrid systems that leverage both organic and inorganic materials. Emerging strategies, such as the incorporation of two-dimensional semiconductors or perovskite nanocrystals into OFET channels, are showing synergistic improvements in mobility, stability, and environmental tolerance. Additionally, scalable printing and additive manufacturing techniques are now being coupled with these high-performance materials to create flexible, large-area electronic platforms that were previously impractical with conventional fabrication methods.

The future of OFETs lies at the intersection of materials innovation, device engineering, and system-level integration. Continued efforts in stabilizing high-mobility semiconductors, controlling interface phenomena, and achieving reproducible device fabrication will be essential for transitioning OFETs from laboratory demonstrations to commercial applications. With these advances, OFETs are poised not only to complement existing silicon technologies but also to enable entirely new paradigms in bioelectronics, wearable computing, and energy-efficient neuromorphic circuits, marking a transformative step in flexible and multifunctional electronics.

### 4.3. Neuromorphic Device

**Figure 15:**
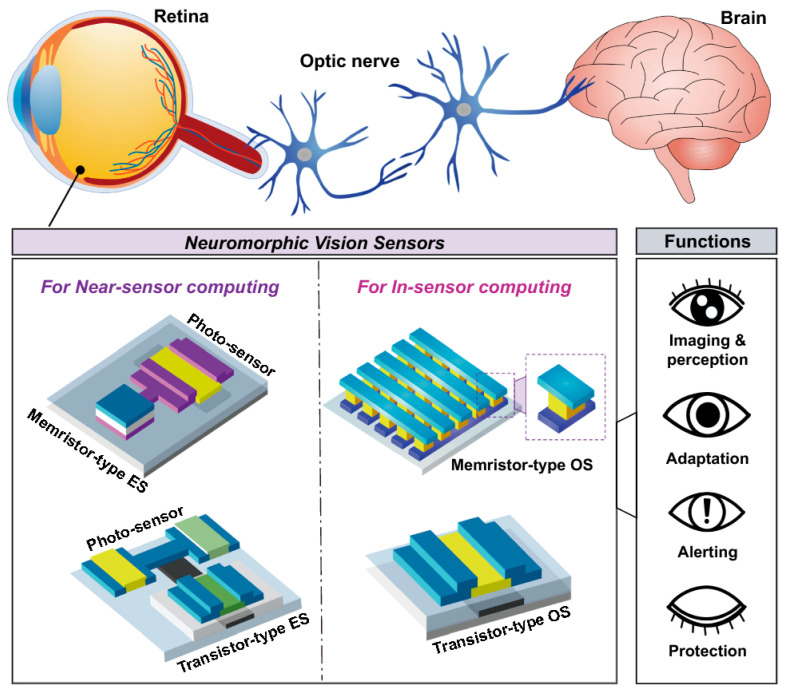
Neuromorphic vision sensors inspired by biological systems, including optoelectronic synaptic circuits (OSCs) that function in a near-sensor computing configuration, and optoelectronic synapses (OSs) that operate within an in-sensor computing framework. Reprinted figure with permission from ref. [80]. Copyright (2022) Springer Nature.

The human visual system, composed of the retina, optic nerve, and visual cortex, serves as a rich biological blueprint for designing neuromorphic vision sensors that integrate sensing and computation. The retina itself is a highly parallel and energy-efficient processor, capable of extracting features such as motion, contrast, and temporal changes before transmitting signals through the optic nerve to higher cortical areas. Inspired by this architecture, neuromorphic vision systems aim to replicate both the sensing and preprocessing capabilities of biological vision in electronic hardware. As illustrated in Figure 15, these systems are generally classified into near-sensor computing and in-sensor computing architectures.

In near-sensor computing, conventional photosensitive elements, such as photodiodes or phototransistors, capture light intensity and convert it into electrical signals. These signals are then routed to external neuromorphic circuits, often realized using memristor arrays or synaptic transistors, which emulate key features of biological synapses. Memristor-type devices exploit resistive switching to store and modulate synaptic weights, enabling operations like temporal integration and short-term memory. Transistor-based synapses, on the other hand, can implement analog signal processing functions such as gain modulation, adaptive filtering, and nonlinear activation. By situating computation near the sensor plane, this architecture reduces latency and lowers the energy cost of transmitting large volumes of raw data to distant processors.

In in-sensor computing architectures, computation is embedded directly within each optical pixel. Devices such as photonic memristors, phototransistors, or hybrid optoelectronic synapses combine light detection and analog signal processing within a single compact structure. For instance, phototransistor-based pixels can respond to varying light intensities with adjustable current outputs that encode temporal and spatial information, while memristive photonic pixels can retain a memory of recent illumination patterns, enabling feature extraction such as edge detection or motion sensing in real time. This tight integration effectively reproduces the retina’s preprocessing functionality, where only salient visual information is transmitted downstream, significantly alleviating data transfer bottlenecks [81].

#### 4.3.1. Device Architectures and Analysis of Neuromorphic Computing

**Figure 16:**
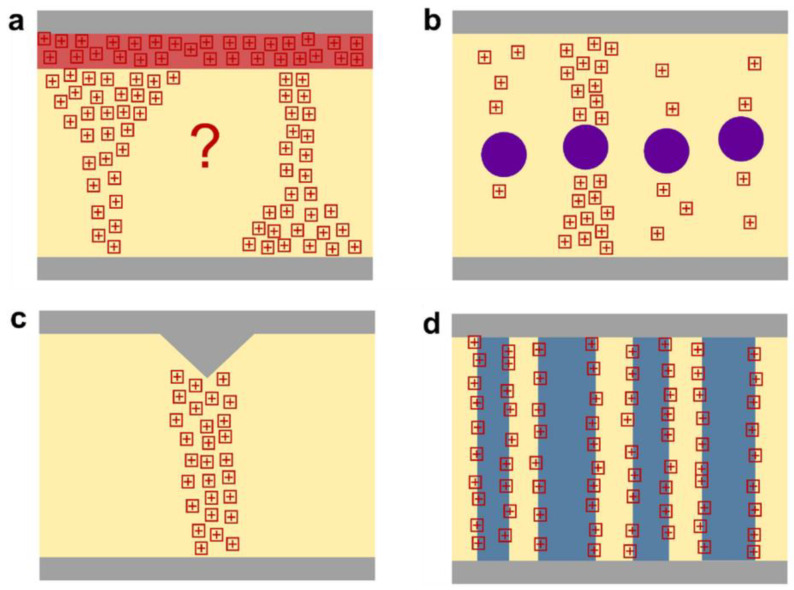
Schematics of representative approaches to explain or control resistive switching mechanisms. Gray regions indicate electrodes, the pale-yellow area denotes the switching layer, and plussed squares represent oxygen vacancies. (**a**) Formation of an oxygen-vacancy-rich region (e.g., TiO_x_) enhances switching. (**b**) Embedded Pt nanoparticles locally strengthen the electric field to guide filament growth. (**c**) Nanoindentations in the top electrode concentrate the field for controlled filament formation. (**d**) Vertically aligned HfO_x_–CeO_x_ nanocomposite films direct filaments along grain boundaries. This figure is adapted from ref. [82] (reproduced under terms of the CC-BY license).

As illustrated in Figure 16, neuromorphic devices can be broadly categorized into two-terminal and three-terminal architectures, each offering distinct functional advantages. Two-terminal devices, including memristors and resistive random-access memory (ReRAM), modulate conductance through filamentary or interfacial redox reactions [82]. Their structure typically consists of a metal–insulator–metal (MIM) stack, where conductance G depends on the migration of ions or defects. The state-dependent current–voltage (I–V) behavior can be obtained with G(V,t) varying dynamically as ions drift under the applied bias. These devices naturally support high-density crossbar arrays, allowing scalable synaptic networks with ultrafast learning dynamics [14,83].

Three-terminal devices, such as electrolyte-gated synaptic transistors and ferroelectric transistors, introduce a gate electrode that modulates the channel conductance through an intermediate dielectric, electrolyte, or ferroelectric layer. This structure enables analog weight tuning and reduced interference between neighboring cells. Ionic migration within the gate dielectric (e.g., ion gel, polymer electrolyte) allows time-dependent modulation of $V_{th}$, effectively emulating synaptic plasticity.

Architectural choices—vertical vs. planar structures, crossbar vs. transistor-type arrays—determine key system-level parameters such as signal latency, energy consumption, and learning linearity. Vertical memristor arrays provide ultrahigh integration density, whereas planar synaptic transistors enable analog weight control and compatibility with flexible substrates. Hybrid 2D/3D arrays combining these architectures are now emerging for multilayer neural networks [84,85].

#### 4.3.2. Materials and Mechanism Model for Neuromorphic Devices

**Figure 17:**
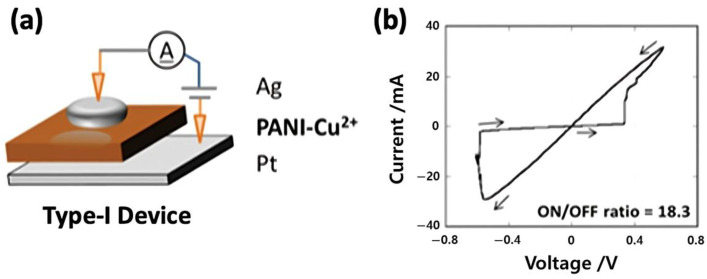
(**a**) Ag/PANI-Cu^2+^/Pt switching device architecture, showing switching between high conductance (ON) and low conductance (OFF) states. (**b**) I–V response of the device measured at a 1 Hz sweep rate. This figure is adapted from ref. [86] (reproduced under terms of the CC-BY license).

A wide range of organic and polymeric semiconductors have been explored for memristor fabrication owing to their tunable electronic structures, low-temperature solution processability, and compatibility with flexible or unconventional substrates. Among polymeric systems, Redox-active conducting polymers such as polyaniline (PANI) have also been widely utilized due to their ability to reversibly modulate oxidation states under an applied electric field (See Figure 17a). Figure 17b demonstrates switching between high-conductance (ON) and low-conductance (OFF) states. PANI-based memristors can exhibit synaptic-like plasticity—including short-term and long-term memory behaviors—by controlling the persistence of the ON state through pulsed biasing. Additionally, these devices often display quantized conductance levels, which are attributed to the formation and rupture of nanoscale metallic filaments within the doped PANI matrix, offering pathways toward highly analog and biologically inspired computing architectures [86]. Poly(3-hexylthiophene) (P3HT) is one of the most extensively studied materials. Its well-defined π–π stacking, semicrystalline morphology, and balanced charge-transport properties facilitate stable resistive switching through mechanisms such as charge trapping, ion migration, and interface dipole modulation. P3HT-based memristors have demonstrated reproducible ON/OFF ratios, low-voltage operation, and sufficient endurance for emerging low-power neuromorphic applications [87,88]. 

Electrophysiological manifestations of short-term plasticity (STP) are frequently observed in experimental studies, yet their significance in neural computation is often underappreciated. A comprehensive understanding of brain function requires examining the dynamic behavior of neural systems and their contribution to information processing. With advances in theoretical frameworks such as statistical physics, nonlinear dynamics, and complex systems, computational models of STP have become increasingly refined. During the 1990s, phenomenological descriptions of STP were developed to capture postsynaptic currents shaped by both paired-pulse facilitation (PPF) and paired-pulse depression (PPD). These modeling approaches consistently demonstrated that synaptic transmission dynamics generate intricate patterns of both regular and irregular network activity. Key factors—including vesicle availability, presynaptic Ca^2+^ concentration, and neurotransmitter release probability—critically determine postsynaptic responses.

A simplified differential model for STP can be expressed as follows [89]:

(10) $$\frac{dx}{dt}=\frac{1-x}{\tau_{D}}-ux\;\delta \left(t-t_{sp}\right)$$

Equation (10) models synaptic depression, representing neurotransmitter depletion. Here, x denotes the normalized vesicle pool reflecting synaptic efficacy, and u represents the normalized release probability. The delta function, $\delta (t-t_{sp})$, captures the timing difference between the current time t and the spike release time $t_{sp}$. The time constants $\tau_{D}$ correspond to the recovery of depression and facilitation toward their baseline values. By adjusting these time constants, the model can phenomenologically reproduce a variety of short-term synaptic behaviors. This framework serves as a foundational reference for the design of STP-inspired devices and neuromorphic hardware.

#### 4.3.3. Synaptic Functions and Charge Transport Mechanisms

Neuromorphic devices are engineered to emulate the information processing and storage functions of biological synapses, serving as the fundamental building blocks of artificial neural networks. These devices are capable of reproducing key synaptic behaviors, including short-term plasticity (STP), long-term potentiation (LTP), and spike-timing-dependent plasticity (STDP), which are essential for learning and memory in biological systems. The realization of these synaptic functions relies on the precise modulation of the device conductance, which acts as an analog or quasi-analog weight in neural computation. By dynamically adjusting conductance states in response to electrical stimuli, memristive and transistor-based neuromorphic devices can encode temporal and spatial correlations of input signals, thereby performing in-memory computation without the need for separate memory and processing units.

The underlying charge transport mechanisms are central to achieving these synaptic behaviors. In filamentary memristors, electrical conduction occurs through nanoscale conductive filaments formed via ion migration or localized redox reactions. The formation, growth, and dissolution of these filaments under applied voltage result in non-linear and hysteretic current–voltage responses that directly mimic synaptic plasticity. In phase-change devices, the modulation of electronic conduction arises from reversible transitions between amorphous and crystalline phases, accompanied by substantial changes in resistivity. Oxide-based memristors rely on the redistribution of oxygen vacancies or other ionic defects, where the interaction between mobile ions and electronic carriers produces complex, history-dependent conductance dynamics. The coexistence of ionic and electronic transport pathways enables devices to exhibit both volatile and non-volatile memory behaviors, corresponding to short-term and long-term synaptic effects, respectively.

Device architecture, material composition, and external operating conditions critically influence these charge transport phenomena. Factors such as the thickness and stoichiometry of the active layer, the choice of electrode materials, and the applied voltage waveform determine the formation kinetics of conductive filaments, ion mobility, and defect migration. Temperature effects further modulate ion diffusion and phase stability, thereby impacting device endurance, switching speed, and energy efficiency. The interplay between these physical parameters and the resulting conductance modulation underpins the high fidelity of synaptic emulation, enabling complex signal integration, temporal coding, and adaptive learning in neuromorphic systems. Understanding these mechanisms at both the material and device levels is crucial for optimizing synaptic functionality and for the development of scalable, energy-efficient artificial neural networks that closely replicate the computational capabilities of biological brains.

#### 4.3.4. Applications and System-Level Integration

Neuromorphic devices are increasingly being integrated into crossbar neural networks, reservoir computing systems, and spiking neural networks (SNNs). Their ability to perform multiply–accumulate (MAC) operations directly in hardware has enabled on-chip learning and inference at power levels several orders of magnitude below those of conventional GPUs [90].

Recent studies demonstrate large-scale integration of memristive arrays (10^4^–10^6^ devices) capable of pattern recognition, adaptive signal processing, and temporal sequence learning. Organic synaptic transistors, operating at sub-1 V and consuming picojoules per event, offer unique compatibility with flexible, wearable, and bio-interfaced systems. Integration with flexible substrates and soft neural interfaces could pave the way toward neuromorphic prosthetics and biohybrid computing, bridging artificial intelligence and biological cognition. In the future, advances in material stability, stochastic tolerance, and circuit-level co-design will be critical to achieving truly scalable neuromorphic systems capable of cognitive computation. The synergistic integration of organic, ferroelectric, and ionic materials will likely define the next frontier of adaptive, low-power artificial intelligence hardware.

### 4.4. Memristor

A memristor, or memory resistor, is a two-terminal passive electronic device whose resistance can be modulated based on the history of applied voltage or current. It exhibits non-volatile memory behavior, retaining its resistance state even in the absence of power. Owing to its simple structure, high scalability, and low energy consumption, the memristor has attracted significant attention for applications in neuromorphic computing, resistive random-access memory (RRAM), and in-memory computing. The resistance switching in memristors generally arises from mechanisms such as ionic migration, redox reactions, or filament formation within the insulating or semiconducting layer. These mechanisms allow reversible transitions between a high-resistance state (HRS) and a low-resistance state (LRS), enabling the emulation of synaptic functionalities in artificial neural networks.

The basic architecture of a memristor comprises three primary components. The top electrode (TE) serves as one terminal of the device and is typically fabricated from metals such as platinum, gold, or titanium nitride. The active layer, located between the electrodes, is an insulating or semiconducting material, often an oxide (e.g., HfO_2_ or TiO_2_) or an organic semiconductor, where resistance switching occurs through ionic migration or conductive filament formation. The bottom electrode (BE) forms the second terminal and is often grounded in circuit configurations.

**Figure 18:**
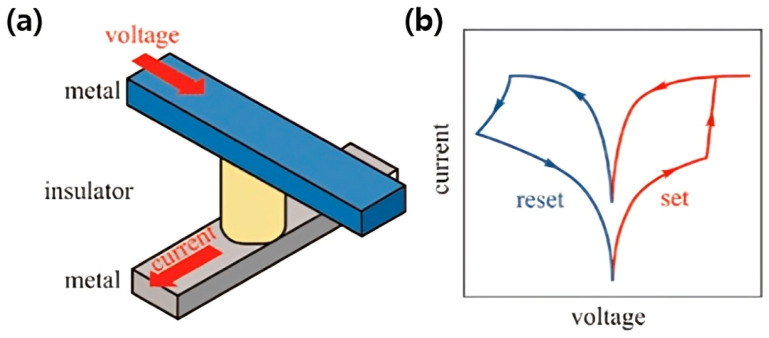
(**a**) Schematic of a memristor featuring a metal/insulator/metal sandwich structure. (**b**) The typical I–V characteristics exhibit a hysteresis loop under voltage sweeping, demonstrating reversible modulation of resistance. This figure is adapted from ref. [91] (reproduced under terms of the CC-BY license).

Figure 18 illustrates a filamentary memristor structure. Under an applied voltage, a conducting filament forms within the insulating layer, creating a low-resistance pathway between the top and bottom electrodes. The top electrode is connected to the applied voltage, while the bottom electrode is grounded. The insulator or semiconductor layer acts as the medium for filament formation and dissolution, enabling resistive switching. By controlling the applied voltage, the filament can be either grown, corresponding to the SET operation, or ruptured, corresponding to the RESET operation, thereby switching the memristor between LRS and HRS. This structure exemplifies a typical two-terminal memristor with filamentary switching, which is commonly employed in RRAM devices and neuromorphic systems.

#### 4.4.1. Switching Mechanisms

The resistive switching behavior in memristive devices originates from several physical and chemical mechanisms, each contributing to the modulation of device resistance. One of the most widely studied mechanisms is conductive filament formation and rupture, where localized paths of high conductivity form within the insulating or semiconducting layer under an applied voltage. The creation and dissolution of these filaments enable reversible transitions between high-resistance and low-resistance states, which are essential for non-volatile memory operation. Redox-based switching is another prominent mechanism, involving the reversible oxidation and reduction of ions or defect sites within the active layer, which alters the electronic conduction pathways. Phase-change mechanisms, observed in chalcogenide materials, rely on the reversible transformation between amorphous and crystalline states, accompanied by significant changes in electrical resistivity. Ionic drift, driven by electric fields, also plays a critical role by redistributing mobile ions within the device, thereby modulating the local conductivity.

The interactions between mobile ions and electronic charges lead to the characteristic memristive hysteresis observed in current–voltage measurements. This hysteresis is a hallmark of memory behavior and reflects the non-linear, history-dependent response of the device. Furthermore, the switching dynamics and operational characteristics of memristors, including switching speed, endurance, retention, and stability, are strongly influenced by external conditions such as temperature, applied voltage, and current. Temperature can affect ion mobility and filament stability, while voltage and current amplitude determine the rate and completeness of the switching processes. A comprehensive understanding of these mechanisms and their dependencies is critical for optimizing memristor performance in applications such as resistive random-access memory, neuromorphic computing, and in-memory computing architectures [92].

#### 4.4.2. Materials, Device Characteristics and Functional Roles in Reservoir Computing

Memristors are emerging as key components in next-generation electronic devices due to their ability to emulate synaptic functions, such as short-term and long-term plasticity, with low energy consumption. While traditional memristors often rely on inorganic oxides, organic and polymeric materials have gained considerable attention because of their solution processability, mechanical flexibility, and tunable electronic properties. For example, conjugated polymers, characterized by alternating single and double bonds along their backbone, enable efficient charge transport and support resistive switching through charge trapping or ion migration. A well-known example is poly(3-hexylthiophene) (P3HT), which has demonstrated stable and reproducible switching behavior. Other conjugated polymers, including polythiophenes, polyfluorenes, and donor–acceptor copolymers (e.g., diketopyrrolopyrrole (DPP)- or naphthalene diimide (NDI)-based polymers), offer tunable energy levels and film morphologies, allowing the engineering of device performance such as ON/OFF ratio, switching speed, and endurance.

Redox-active polymers, such as polyaniline (PANI) and poly(3,4-ethylenedioxythiophene) (PEDOT), can undergo reversible oxidation-reduction reactions, providing intrinsic mechanisms for memristive behavior. In these materials, the modulation of oxidation states allows dynamic adjustment of the device conductance, which can mimic synaptic weight updates in neuromorphic circuits. For example, PANI-based memristors have exhibited short-term and long-term plasticity, as well as quantized conductance states, suggesting the formation of filamentary conduction paths within the polymer layer.

The resistive switching mechanisms described in Section 4.4.1 fundamentally govern the operational capabilities of memristors in reservoir computing (RC) systems. The non-linear and history-dependent behavior arising from conductive filament formation and rupture, redox reactions, phase-change processes, and ionic drift enables memristors to act as dynamic nodes capable of transforming temporal input signals into high-dimensional representations. Such non-linear transformations are essential for the reservoir to efficiently encode temporal features, facilitating subsequent readout by simple linear learning algorithms.

Key device characteristics, including switching speed, endurance, retention, and the degree of hysteresis, directly influence the richness and complexity of the reservoir dynamics. For instance, fast switching speeds allow the reservoir to process rapidly varying input signals, while long retention times ensure that past inputs continue to influence current responses, embodying the fading memory property critical for temporal computation. Hysteresis and non-linearity provide the diversity of responses required for complex temporal pattern recognition, and controlled variability among devices can be exploited to introduce heterogeneity into the reservoir, enhancing computational richness.

Furthermore, device-level characteristics influence system-level performance in memristor-based RC architecture. Variability in switching thresholds, conductance states, and response times can serve as a source of functional diversity, enabling reservoirs with heterogeneous dynamics that improve the separability of temporal input patterns. Such features highlight a critical departure from conventional electronic devices: rather than minimizing variability, memristor-based RC systems leverage it to enhance computational capability. Collectively, the interplay between intrinsic switching mechanisms, device operational characteristics, and environmental conditions establishes the foundation for high-performance memristor-based RC systems, providing a seamless transition from physical device behavior to practical neuromorphic computation.

#### 4.4.3. Roadmap of the Historical Development of Memristor-Based RC

**Figure 19:**
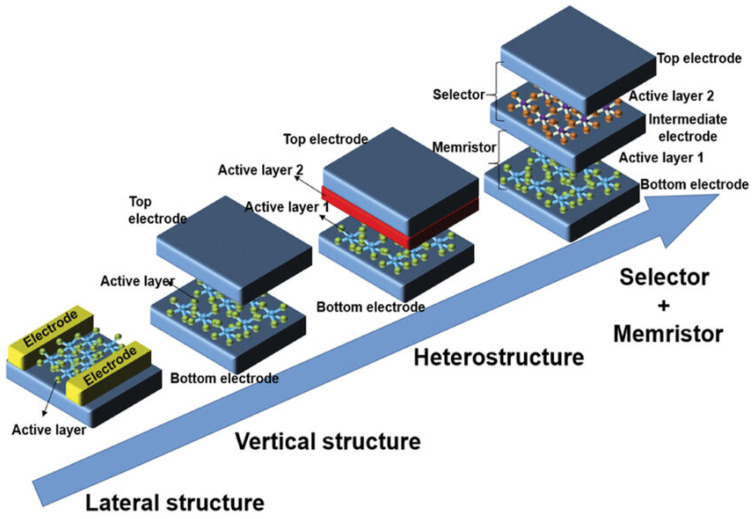
Schematic illustration of various memristor device architectures, progressing from lateral to vertical structures, heterostructures, and integrated selector–memristor configurations. Each design consists of electrodes and one or more active layers, enabling diverse switching behaviors and improved device performance. This figure is adapted from ref. [93] (reproduced under terms of the CC-BY license).

Figure 19 illustrates the evolution of device architectures from a simple lateral structure to a more complex vertical heterostructure and, finally, to an integrated selector + memristor configuration. The lateral structure consists of a single active layer sandwiched between two electrodes placed side by side. The vertical structure stacks the active layer(s) between top and bottom electrodes, enabling more compact and efficient charge transport. In the heterostructure, multiple active layers are incorporated with intermediate electrodes, allowing enhanced functionality and tunable electrical characteristics. The final configuration combines a selector with a memristor, integrating multiple layers and components to achieve controlled switching behavior and improved performance for advanced memory or neuromorphic applications.

In reservoir computing (RC), the reservoir nonlinearly maps temporal inputs into a high-dimensional space, enabling efficient feature extraction via simple learning algorithms. Any dynamical system with rich dynamics and fading memory can serve as a reservoir, including electrical circuits, photonic, spintronic, mechanical, biological, and material-based systems [94]. Physical reservoirs offer rapid processing with low learning overhead, positioning memristor-based RC as a promising hardware paradigm [95]. Given their simple structure, biomimetic properties, and high integration density, memristor-based RC systems have expanded rapidly.

Memristors—the fourth fundamental passive element—exhibit a resistance that varies nonlinearly with the applied voltage or current. First theorized by Chua in 2003 [96], nanoscale memristors with crossbar architectures offer high-density integration, making them promising for in-memory and computing-in-memory applications. Their dynamic memory behavior allows them to emulate synapses with continuously adjustable weights and neurons with tunable firing thresholds, enabling applications in modeling, clustering, real-time coding, and data compression [94]. Memristors are classified as volatile or nonvolatile based on the retention of their low-resistance state. Nonvolatile devices exhibit long-term memory (LTM), reinforcing resistance states with prior excitation, whereas volatile devices relax spontaneously, mimicking short-term memory (STM) and integrate-and-fire neuron behavior. Both types can display nonlinear fading memory, in which resistance depends on recent inputs rather than distant past signals—a critical feature for temporal processing. This allows a single memristor to provide both nonlinear dynamics and fading memory, making it an ideal reservoir element.

#### 4.4.4. Applications and Integration in Circuits

Memristor-based reservoir computing (RC) has demonstrated considerable potential across a wide range of information processing tasks, leveraging the inherent non-linearity, memory effects, and high-dimensional mapping capabilities of memristive devices. One prominent application is temporal signal processing, where memristor reservoirs efficiently capture and transform time-varying input signals, enabling tasks such as speech recognition, gesture classification, and time-series prediction. The ability of memristors to exhibit short- and long-term memory effects allows the reservoir to encode both recent and past inputs, providing a robust platform for sequential and temporal data analysis.

In addition to temporal processing, memristor-based RC systems have been explored for pattern recognition and classification tasks in neuromorphic computing. By exploiting device-level variability and heterogeneous switching behaviors, reservoirs can generate diverse internal representations of input patterns, improving separability and classification performance. Applications in edge computing and low-power artificial intelligence are particularly promising, as memristor arrays integrate memory and computation in a compact form factor, significantly reducing energy consumption and latency compared to conventional von Neumann architectures.

Beyond classical RC tasks, memristor-based systems have been applied to dynamic control, forecasting, and real-time signal filtering. For example, in robotic control and autonomous systems, memristor reservoirs can process sensor data in situ, enabling adaptive responses without relying on centralized computation. Furthermore, the scalability of memristor arrays allows the construction of large reservoirs capable of handling complex, high-dimensional data streams, opening avenues for advanced machine learning and neuromorphic applications. Collectively, these developments highlight the versatility and promise of memristor-based RC as a hardware-efficient, adaptive computing paradigm that bridges device physics and functional computation in a wide range of practical scenarios.

## 5. Emerging Directions and Perspectives

The field of polymeric semiconductors has witnessed significant advancements, driven by the demand for sustainable, flexible, and high-performance electronic devices. This chapter delves into three pivotal areas: degradable and sustainable polymeric semiconductors, hybrid and two-dimensional (2D) polymer semiconductor systems, and the challenges and future directions in polymeric semiconductors.

### 5.1. Emerging Directions Toward Next-Generation Polymeric Semiconductors

The field of polymeric semiconductors has undergone rapid evolution over the past decade, driven by the demand for sustainable, flexible, and high-performance electronic devices. While conventional conjugated polymers have enabled numerous applications in organic electronics, emerging directions focus on addressing the limitations of these materials and exploring new functionalities. These directions encompass molecular design strategies, hybrid material integration, and environmentally conscious approaches, all aimed at enabling next-generation electronic technologies.

One major emerging trend is the development of degradable and sustainable polymeric semiconductors. Researchers are increasingly designing conjugated polymers with cleavable linkages, such as ester, imine, or disulfide bonds, which allow controlled degradation under mild environmental conditions. Such materials not only reduce the ecological impact of electronic waste but also open possibilities for transient electronics and fully recyclable devices. In addition, green synthesis approaches—including the use of biomass-derived monomers, solvent-free polymerizations, and recyclable dopants—have demonstrated that high-performance electronic properties can be maintained while minimizing environmental footprints. This convergence of performance and sustainability represents a paradigm shift in polymer electronics, aligning material development with global sustainability goals.

Another key direction involves hybrid and two-dimensional (2D) polymer semiconductor systems. By combining polymeric semiconductors with 2D materials such as graphene, MoS_2_, or transition metal dichalcogenides, researchers have achieved significant improvements in charge transport, mechanical robustness, and device stability. Hybrid architectures, including polymer–perovskite or polymer–metal oxide composites, enable enhanced energy-level alignment and multifunctional properties for applications in photovoltaics, photodetectors, and flexible electronics. These hybrid systems exemplify a trend toward multifunctional, high-efficiency platforms that transcend the limitations of traditional polymer semiconductors.

Finally, the field is increasingly focusing on challenges and future opportunities. Key issues include improving environmental and operational stability, maintaining high mobility under mechanical stress, and scaling up sustainable synthesis and fabrication techniques. Future research is expected to integrate molecular-level design, advanced processing methods such as additive manufacturing, and computational approaches like machine learning for accelerated materials discovery. By addressing these challenges, emerging research directions aim to establish polymeric semiconductors as versatile, sustainable, and high-performance materials for next-generation electronic devices.

### 5.2. Degradable and Sustainable Polymeric Semiconductors

Degradable and sustainable polymeric semiconductors are emerging as a critical class of materials for environmentally friendly electronics. Traditional electronic materials often rely on nonrenewable resources and generate persistent waste, posing long-term ecological risks. In contrast, degradable polymeric semiconductors are designed to break down into non-toxic components under specific environmental conditions, such as exposure to moisture, light, or enzymatic action. Their molecular architecture typically incorporates hydrolyzable bonds, such as ester, anhydride, or imine linkages, which allow controlled chemical decomposition while retaining semiconducting properties during device operation.

From a scientific perspective, the charge transport in degradable polymeric semiconductors remains fundamentally similar to that in conventional conjugated polymers, dominated by the hopping of charge carriers between π-conjugated segments. The key difference is that the backbone and side-chain chemistries are tailored to enable both electronic functionality and biodegradability. By optimizing molecular weight, crystallinity, and chain packing, researchers can balance charge mobility and degradation kinetics. The inclusion of green solvents and renewable feedstocks, such as polylactic acid or cellulose derivatives, further enhances sustainability without compromising device performance. Applications of these materials span transient electronics, such as biodegradable sensors for medical implants or environmental monitoring, where the device safely decomposes after fulfilling its function. The challenges remain in achieving high charge mobility and long-term operational stability while maintaining complete environmental degradability [97,98,99].

### 5.3. Hybrid and 2D–Polymer Semiconductor Systems

Hybrid polymer semiconductors combine conjugated polymers with inorganic or two-dimensional (2D) materials, offering unique opportunities to tailor electronic, optical, and mechanical properties. 2D materials such as graphene, transition metal dichalcogenides (TMDs), and black phosphorus possess exceptional carrier mobilities, tunable bandgaps, and atomically thin profiles. When integrated with polymers, they create heterostructures that leverage the solution processability, flexibility, and chemical tunability of polymers alongside the superior electronic performance of 2D layers. In hybrid systems, charge transfer occurs at the polymer/2D material interface, which can be tuned through energy level alignment, surface functionalization, or controlled morphology. For instance, graphene oxide combined with a conjugated polymer can improve electron transport pathways, while TMD nanosheets can enhance photoresponse and exciton dissociation in polymer-based photodetectors. The hybridization not only enhances device performance but can also introduce novel functionalities, such as ambipolar transport, broadband photodetection, or mechanically resilient flexible electronics.

Fabrication techniques for these systems range from layer-by-layer deposition, solution blending, and in situ polymerization to more advanced approaches such as van der Waals assembly of 2D sheets. The challenge lies in achieving uniform dispersion, strong interfacial contact, and defect-free films without degrading the intrinsic properties of either component. Optimizing these parameters requires a deep understanding of molecular interactions, crystallization behavior, and nanoscale morphology, often guided by advanced characterization methods like atomic force microscopy, X-ray scattering, and electron spectroscopy [100,101].

### 5.4. Challenges and Future Directions in Polymeric Semiconductors

Despite the progress made, several challenges remain in the development of polymeric semiconductors. One of the primary issues is the trade-off between processability and performance. While solution-processable polymers offer advantages in terms of fabrication scalability and cost, they often exhibit lower charge carrier mobility compared to their inorganic counterparts. To address this, strategies such as molecular engineering, blending, and the incorporation of nanostructures are being explored. Molecular engineering involves the design of polymer backbones and side chains to enhance π–π stacking interactions and crystallinity, thereby improving charge transport. Blending different polymers can lead to phase separation, creating nanoscale domains that facilitate charge carrier movement. Additionally, the incorporation of nanostructures, such as nanoparticles or nanofibers, can provide pathways for charge carriers and enhance the overall conductivity.

Another challenge is the stability of polymeric semiconductors under operational conditions. Environmental factors such as humidity, oxygen, and light can degrade the performance of these materials. To mitigate this, encapsulation techniques and the development of intrinsically stable polymers are being investigated. Looking forward, the integration of artificial intelligence (AI) and machine learning (ML) into the design and optimization of polymeric semiconductors holds great promise. These technologies can accelerate the discovery of new materials by predicting the properties of polymers based on their chemical structure. Furthermore, AI and ML can be employed to optimize processing conditions and device architectures, leading to enhanced performance and reliability. Ultimately, the field of polymeric semiconductors is poised for significant advancements, driven by innovations in material design, processing techniques, and computational tools. Overcoming the existing challenges will pave the way for the development of sustainable, high-performance electronic devices that can meet the demands of future technologies. The convergence of sustainable materials science, nanoscale engineering, and computational design is expected to drive the next generation of polymeric semiconductors, enabling flexible, biodegradable, and high-performance electronic systems that align with environmental sustainability goals [102,103].

## 6. Conclusions

Polymeric semiconductors have progressed from simple conductive polymers to high-performance donor–acceptor systems with tunable electronic structures, enhanced charge-carrier mobility, and solution-processable device compatibility. Their electronic properties are governed by tightly coupled factors—molecular packing, energetic disorder, mixed ionic–electronic transport, and thin-film morphology—while advances in in-situ characterization and precision processing are enabling more predictive control over these parameters. Interface engineering, molecular doping, and optimized energy-level alignment continue to improve device efficiency and stability across applications including organic photovoltaics, field-effect transistors, memristors, and neuromorphic circuits. Future progress will rely on elucidating quantitative structure–property relationships, particularly the roles of dynamic disorder, polaron transport mechanisms, and interfacial charge transfer. Machine-learning-guided polymer design, high-throughput synthesis, and scalable patterning techniques offer promising routes for accelerating material discovery and device integration. Remaining challenges—such as operational stability, reproducibility, and compatibility with large-area fabrication—must be addressed to enable reliable deployment. With these advances, polymeric semiconductors are positioned to serve as a foundational platform for next-generation flexible, low-power, and sustainable electronic systems.

## Figures and Tables

**Table 1:** Electrical conductivities of polymeric materials.

Polymer	Doped Conductivity [S/cm]	Undoped Conductivity [S/cm]
polyacetylene	$10^{6}-10^{8}$	$10^{-2}-10^{-8}$
polypyrrole	$10^{2}-10^{4}$	$10^{-8}$
polythiophene	$10^{2}$	$10^{-8}$
poly(3,4-ethylenedioxythiophene)	$10^{2}-300$	$10^{-8}$
polyaniline	$10^{2}$	$10^{-8}-10^{-10}$
poly(p-phenylene vinylene)	$10^{2}-10^{-3}$	$10^{-8}-10^{-13}$
poly(para-phenylene)	$10^{2}-500$	$10^{-8}$

## Data Availability

No new data were created or analyzed in this study. Data sharing is not applicable to this article.
